# Recent advances in the total synthesis of limonoid natural products: a decade of progress

**DOI:** 10.1039/d6ra08819h

**Published:** 2026-10-22

**Authors:** Wei Chen, Xiaobo Zhou, Dongsheng Yang

**Affiliations:** a College of Life Sciences, Zhuhai College of Science and Technology Zhuhai 519041 China yds@zcst.edu.cn smuchenwei@foxmail.com; b State Key Laboratory of Chemical Oncogenomics, Key Laboratory of Chemical Genomics, Peking University Shenzhen Graduate School Shenzhen 518055 China

## Abstract

Limonoid natural products are highly oxygenated tetranortriterpenoids with broad-spectrum biological activities, yet their compact polycyclic frameworks and multiple contiguous stereocentres render them formidable targets for total synthesis. This review summarizes recent advances in the total synthesis of limonoid natural products from 2017 to 2026. The discussion is organised by the structural classification of limonoids, covering ring-intact limonoids, ring-*seco* limonoids (including ring C-*seco*, D-*seco*, A,D-*seco*, and B,D-*seco* types), rearranged limonoids, and limonoid alkaloids. Emphasis is placed on key strategic disconnections and the emergence of modern methodologies such as photoredox catalysis, C–H activation, metal–hydrogen-atom-transfer-mediated radical cyclisation, and chemoenzymatic approaches.

## Abstract 2

This review summarizes recent advances in the total synthesis of limonoid natural products from 2017 to 2026, organized by the structural classification.

## Graphical abstract

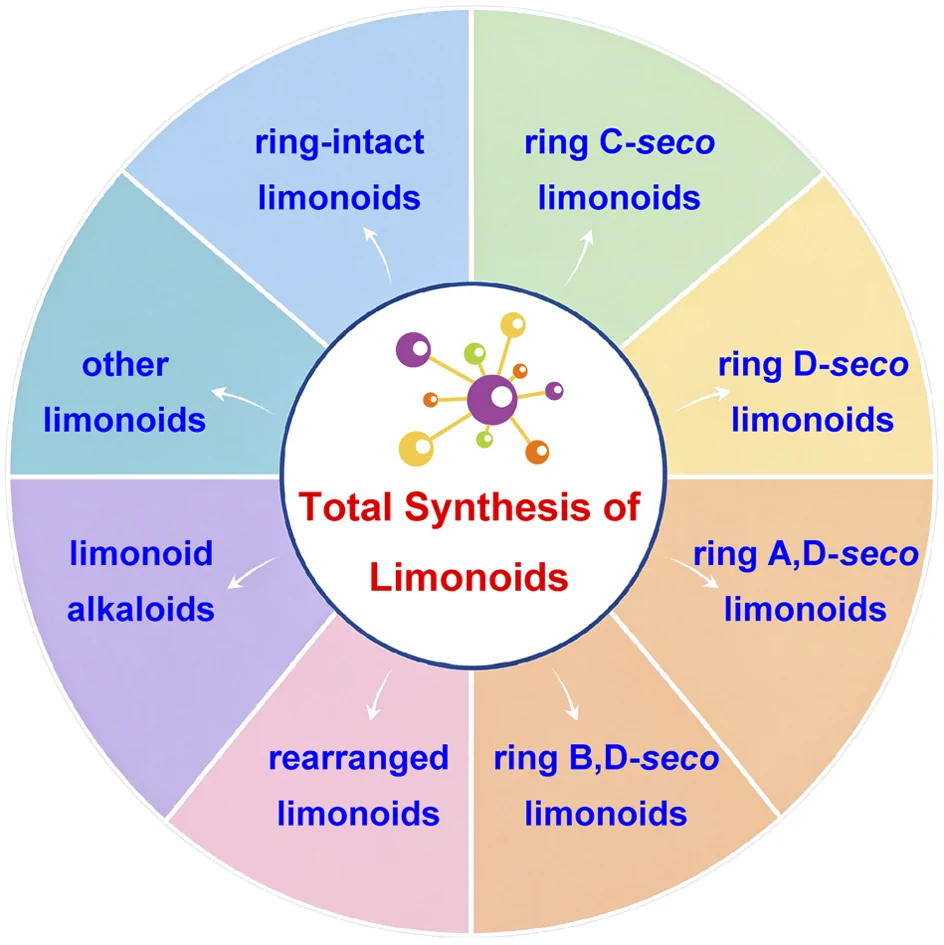

## 1. Introduction

Research on limonoid natural products has a history of nearly two centuries, originating from studies of the characteristic bitterness of citrus fruits.^1^ The term “limonoids” derives from limonin, the first tetranortriterpenoid isolated from citrus bitter principles.^2^ Limonin was first obtained from Washington navel orange by Higby in 1938 (ref. 3) and subsequently identified as the principal bitter component of navel orange juice in 1949.^4^ Early investigations were primarily driven by the commercial need to mitigate the “delayed bitterness” phenomenon that adversely affected the citrus juice and fruit processing industry worldwide.^5,6^ Over the ensuing decades, however, the research interest gradually shifted from bitterness mitigation to the remarkable structural diversity and broad-spectrum biological activities of these compounds, and limonoids have become one of the most intensively studied classes of plant secondary metabolites.^7–9^

In the plant kingdom, limonoids occur predominantly in the Rutaceae and Meliaceae families, with less frequent distribution in *Cneoraceae*, *Ptaeroxylaceae*, and *Harrisonia* sp. of *Simaroubaceae*.^2,10–12^ Chemically, limonoids are highly oxygenated tetranortriterpenoids that either possess the prototypical 4,4,8-trimethyl-17-furanylsteroid skeleton or are formally derived from a precursor bearing this motif.^13,14^ Structurally, limonoids are classified into several major subcategories based on the degree of oxidative modification and skeletal rearrangement of the parent pentacyclic carbon framework (Fig. 1).^13,14^ (i) Ring-intact limonoids retain the unaltered connectivity of the parent androstane-type A/B/C/D ring system with the C17 furan substituent (E ring). (ii) Ring-*seco* limonoids arise from the oxidative cleavage of one or more rings of the parent nucleus, depending on which ring(s) are cleaved; they are further designated as ring A-*seco*, B-*seco*, C-*seco*, D-*seco*, or multiply ring *seco* types. (iii) Rearranged limonoids (including limonoid alkaloids) undergo more complex skeletal transformations, including C–C bond migration, ring contraction or expansion, and novel C–C or C–N/C–O linkage formation. (iv) Degraded limonoids represent structurally simplified derivatives, typically resulting from extensive oxidative degradation of the parent nucleus to yield fused bicyclic γ- or δ-lactone systems.

**Fig. 1:**
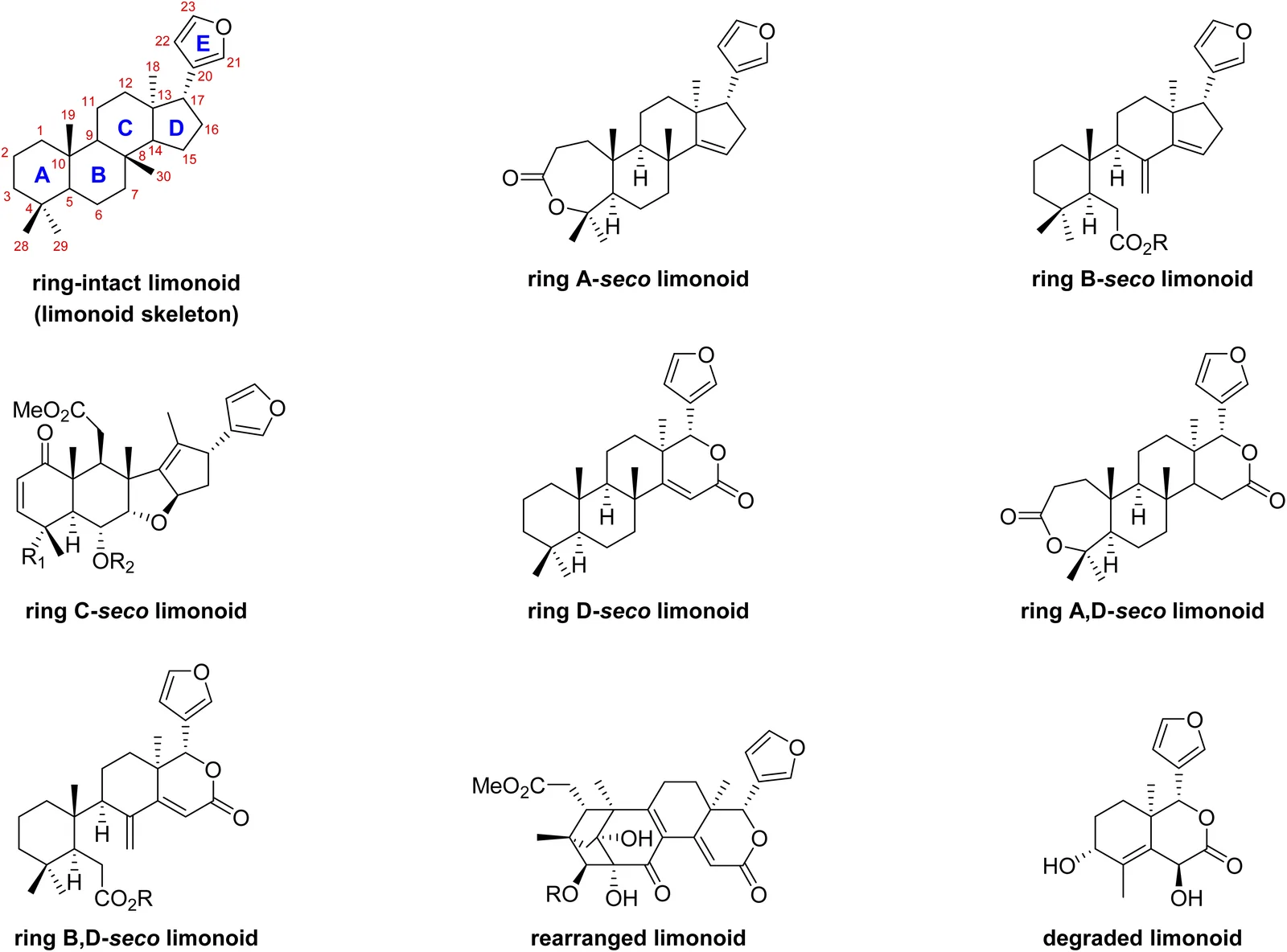
Classification of representative limonoid natural products. The prefix “*seco*” derives from the Latin *secāre* (means to cut).

The biological activities attributed to limonoids are exceptionally broad and have been extensively reviewed.^15–20^ In the agricultural context, limonoids exhibit potent insect antifeedant, insecticidal, and insect growth-regulatory activities against a wide range of agriculturally and medically significant pest species.^21^ In medicinal research, limonoids have demonstrated cytotoxic and anticancer activity against multiple tumour cell lines,^22,23^ together with anti-inflammatory, antioxidant, antimicrobial, antiviral, antimalarial, antiprotozoal, neuroprotective, and metabolic–regulatory activities.^15–20^ The structural complexity and dense oxygenation of limonoids are believed to underpin their ability to interact with diverse biological targets, making them privileged scaffolds for drug discovery and chemical biology research.^13,14^

Despite their profound biological significance and the enormous number of naturally occurring limonoids, the chemical total synthesis of these complex molecules remains a formidable challenge.^24–26^ The principal synthetic obstacles include: (i) the compact, highly oxygenated three-dimensional framework with up to sixteen oxygen atoms densely packed within a relatively small carbon skeleton; (ii) the presence of multiple contiguous stereocenters, often including quaternary centers; (iii) the sensitivity of the furan ring and various oxygenated functionalities to acidic, basic, and oxidative reaction conditions; (iv) the challenge of constructing the characteristic rearranged carbon frameworks in a controlled and stereoselective manner; (v) the limited availability of functionalized starting materials that can be efficiently elaborated to the target skeleton. The historical difficulty of limonoid synthesis is perhaps best exemplified by azadirachtin, the structurally complex ring C-*seco* limonoid from the neem tree (*Azadirachta indica*), whose landmark total synthesis by Ley and co-workers required a 22-year synthetic campaign.^27,28^

The past decade has witnessed substantial progress in the total synthesis of limonoids, driven by the development and application of modern synthetic methodologies. This review provides a comprehensive survey of total synthesis of limonoid natural products reported from 2017 to 2026. To maintain a clear focus, the scope is restricted to full total and formal total synthesis of limonoid natural products. The synthetic studies directed only toward the construction of limonoid carbon skeletons or advanced fragments are excluded.^29–39^ In addition, degraded limonoids are not covered, owing to their distinct structural features and the availability of dedicated reviews on their synthesis.^25,40^

## 2. Biosynthetic pathway of limonoids

Limonoid natural products are tetranortriterpenoids (26-carbon) derived from the universal 5-carbon isoprenoid precursors isopentenyl diphosphate (IPP) and dimethylallyl diphosphate (DMAPP),^41^ as depicted in Fig. 2. Stable isotope labelling and pathway-specific inhibitor studies have established the mevalonate (MVA) pathway as the predominant source of 5-carbon isoprenoid units for limonoid biosynthesis.^42^ IPP and DMAPP undergo sequential head-to-tail condensation catalysed by geranyl diphosphate synthase (GPPS) and farnesyl diphosphate synthase (FPPS) to afford farnesyl diphosphate (FPP).^41,42^ Two molecules of FPP are then reductively coupled by squalene synthase (SQS) to squalene, which is subsequently epoxidised by squalene epoxidase (SQE) to yield 2,3-oxidosqualene, the common precursor of all plant triterpenoids.^41–43^

**Fig. 2:**
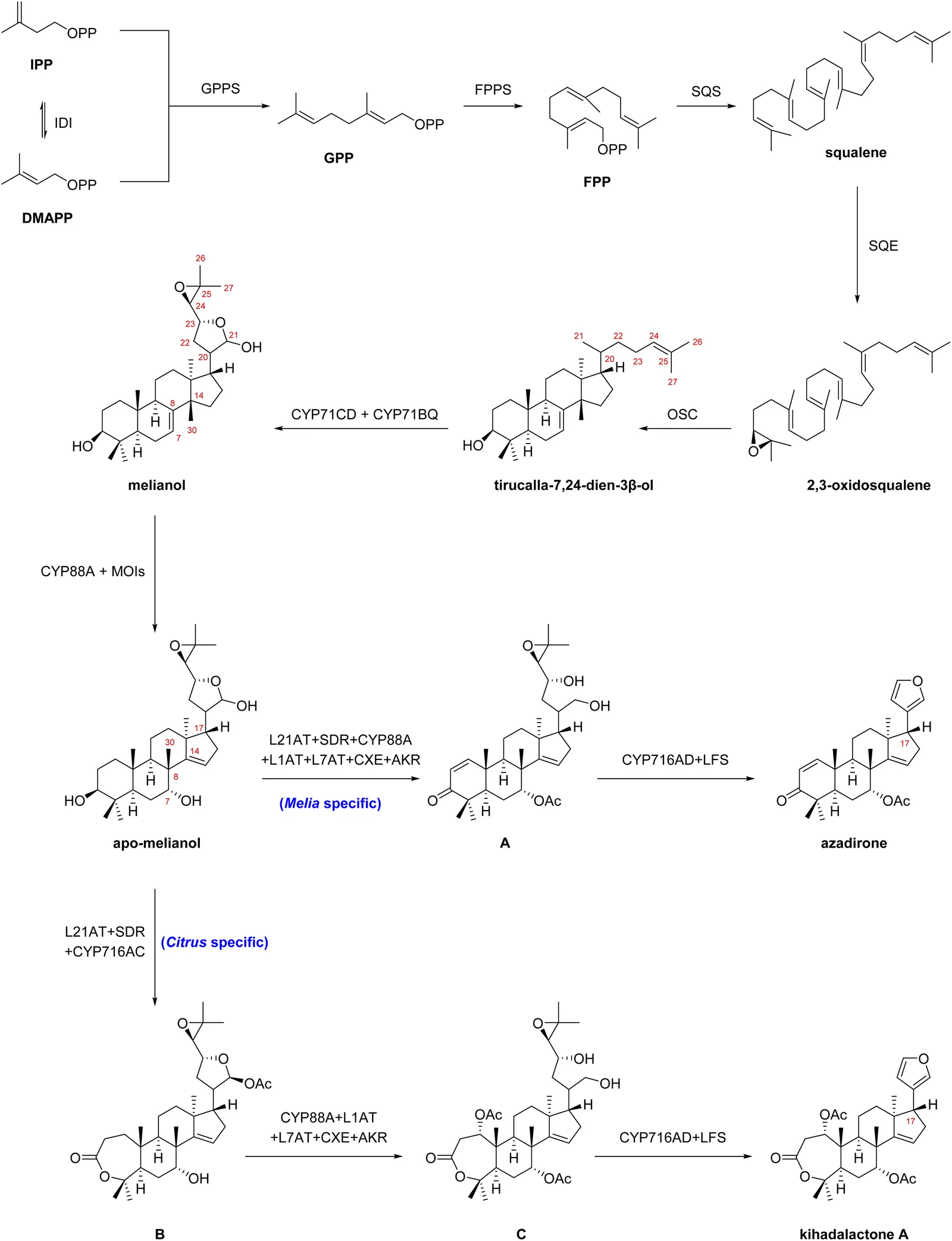
Biosynthetic pathway of limonoid natural products.

The first committed step in limonoid biosynthesis is the cyclisation of 2,3-oxidosqualene by a specialized oxidosqualene cyclase (OSC) to generate the tetracyclic triterpene tirucalla-7,24-dien-3β-ol.^43,44^ Functional orthologues have been characterised from *Azadirachta indica* (AiOSC1), *Melia azedarach* (MaOSC1), and *Citrus sinensis* (CsOSC1), establishing the tirucallane scaffold as the conserved triterpene precursor in both the Meliaceae and Rutaceae families.^45,46^ Subsequently, two co-expressed cytochrome P450 enzymes of the CYP71 family (CYP71CD and CYP71BQ) catalyse three sequential oxidations on the side chain *via* the intermediate dihydroniloticin to afford the first protolimonoid, melianol (30-carbon).^46^ This three-enzyme module (OSC/CYP71CD/CYP71BQ) is highly conserved across both plant families.^43,46^

A hallmark of limonoid biosynthesis is the post-cyclisation skeletal rearrangement that follows melianol.^46,47^ A CYP88A subfamily enzyme (CsCYP88A51 and MaCYP88A108) oxidises the C7/C8 position to generate an unstable epoxide intermediate, which is captured by neofunctionalised sterol isomerases renamed melianol oxide isomerases (MOIs).^46^ The MOIs direct migration of the C30 methyl group from C14 to C8 (the apo-rearrangement), yielding apo-melianol and establishing the canonical limonoid carbon framework.^46^ Thereafter, a suite of tailoring enzymes, including limonoid 21-*O*-acetyltransferase (L21AT), short-chain dehydrogenase/reductase (SDR), limonoid 1-*O*- and 7-*O*-acetyltransferases (L1AT, L7AT), and additional cytochrome P450s, installs multiple oxygenations and acylations across the scaffold.^46^

The conversion of 30-carbon protolimonoids to 26-carbon limonoids entails side-chain cleavage coupled to furan-ring formation. Following deacetylation by a carboxylesterase (CXE),^48^ an aldo-keto reductase (AKR) reduces the open-chain aldehyde; a CYP716AD subfamily enzyme then effects partial side-chain cleavage.^46,48^ Finally, limonoid furan synthase (LFS), a novel 2-oxoglutarate-dependent dioxygenase, catalyses oxidative cleavage and cyclisation, with concomitant loss of the C24–C27 fragment, to construct the signature C17 furan ring and deliver the basal 26-carbon limonoid scaffold.^46,48^ In the Meliaceae, the biosynthetic pathway yields azadirone (ring-intact limonoid), whereas in the Rutaceae, it affords kihadalactone A (ring A-*seco* limonoid).^46^ Each serves as the branchpoint precursor for the downstream diversification of family-specific limonoids.^43,46,48^

## 3. Total synthesis of ring-intact limonoids

### 3.1 Ma's total synthesis of (−)-azadiradione (2025)

Azadiradione, a prototypical ring-intact limonoid, was first isolated from the stem bark of neem tree (*Melia azadirachta* L.) by Lavie and co-workers in 1967.^49,50^ Structurally, it features a densely functionalised 6/6/6/5 tetracyclic carbon framework bearing a furan moiety at C17 and an α,β-unsaturated ketone in the A-ring. Biologically, azadiradione has been identified as a potent activator of heat shock factor 1 (HSF1),^51^ a master regulator of the proteotoxic stress response, and also exhibits insect antifeedant, anti-inflammatory, and anticancer activities.^13,20^ Despite its structural and biological significance, efficient synthetic access to azadiradione has long remained a formidable challenge. The pioneering total synthesis by Corey's group in 1989 required 27 linear steps.^52^

In 2025, Ma's group reported a concise and convergent total synthesis of (−)-azadiradione (17), along with three related ring-intact limonoids (1,2-dihydroazadirone, azadirone, and epoxyazadiradione), in 12–15 steps from the commercially available terpene feedstock sclareolide (Scheme 1).^53^ Their retrosynthetic strategy dissected the tetracyclic framework into two fragments of comparable complexity: a tricyclic aldehyde fragment (10) embodying the A/B-rings and a silyl enol ether fragment (4) incorporating the D/E-rings. The central C-ring, bearing the challenging C8 quaternary stereocenter, was to be forged through a Pd-catalysed intramolecular Heck reaction, while the C12–C13 bond with its adjacent C13 quaternary center would be established *via* a convergent intermolecular aldol coupling.

**Scheme 1:**
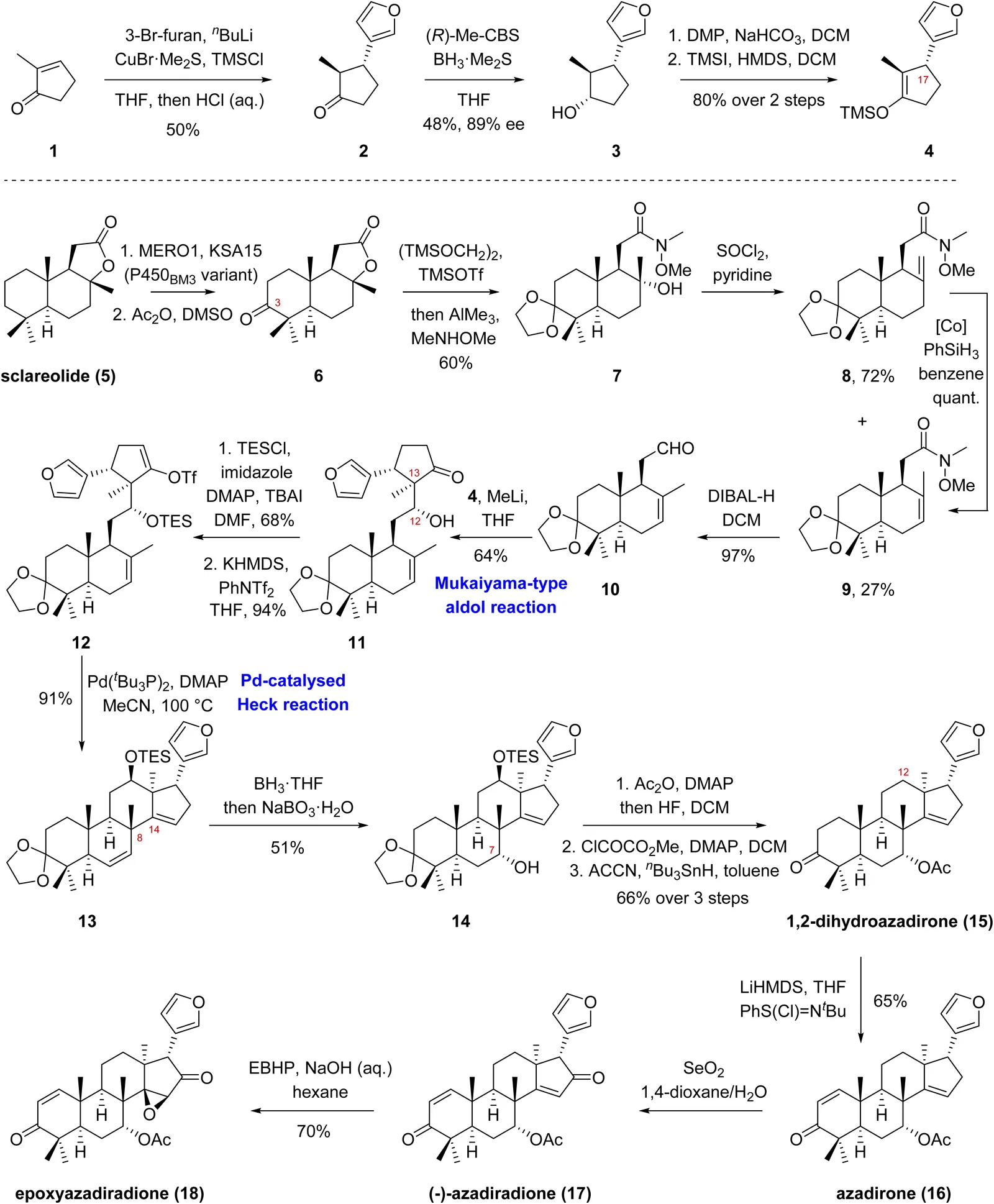
Ma's total synthesis of (−)-azadiradione.^53^

The synthesis commenced with the parallel preparation of two fragments. For the D/E fragment (4), conjugate addition of enone 1 with the cuprate derived from 3-furyl lithium followed by *in situ* deprotection with aqueous HCl furnished ketone 2. Corey–Bakshi–Shibata reduction of 2 and subsequent Dess–Martin oxidation provided the chiral ketone with the desired C17 stereocenter,^54^ which was converted to the thermodynamic silyl enol ether 4 under the action of TMSI and HMDS.^55^ For the A/B fragment (10), selective C–H oxidation of sclareolide (5) at C3 gave compound 6.^56^ Ketal formation and subsequent lactone opening with *N*,*O*-dimethylhydroxylamine were carried out in a one-pot protocol to afford Weinreb amide 7. Dehydration of 7 with SOCl_2_ in pyridine gave *exo*-olefin 8 as the major product, which was isomerized to the desired *endo*-olefin 9 by using Shenvi's method.^57^ Reduction of 9 with DIBAL-H eventually delivered aldehyde 10.

With both fragments in hand, treatment of silyl enol ether 4 with MeLi generated the corresponding lithium enolate, which reacted with aldehyde 10 to give aldol product 11 bearing the desired all-carbon quaternary center at C13. Protection of the C12 hydroxyl with TESCl was followed by conversion to enol triflate 12. The pivotal Pd-catalysed intramolecular Heck reaction of 12 proceeded in quantitative yield by using Pd(^*t*^Bu_3_P)_2_ as catalyst and DMAP as base, constructing the central six-membered C-ring and installing the C8 quaternary stereocenter to afford pentacyclic product 13. This transformation also proceeded in 91% yield on a gram scale.

Chemo- and stereoselective hydroboration-oxidation of 13 furnished alcohol 14 with the desired stereocenter at C7. Acylation of 14 with Ac_2_O and subsequent removal of the ketal and TES groups with HF were carried out in a one-pot protocol. Barton deoxygenation of the C12 hydroxyl provided 1,2-dihydroazadirone (15) in 66% yield over three steps. Mukaiyama dehydrogenation of 15 with LiHMDS and *N-tert*-butyl phenyl sulfinimidoyl chloride afforded azadirone (16) in 65% yield.^58^ Finally, SeO_2_-mediated allylic oxidation of 16 delivered (−)-azadiradione (17),^50^ which could be converted to epoxyazadiradione (18) through a chemoselective epoxidation.^59^

### 3.2 Renata's total synthesis of (−)-azadiradione and (+)-Corey's protolimonoid (2025)

In the same year, Renata's group disclosed a chemoenzymatic total synthesis of (−)-azadiradione (17) in 15 steps (Scheme 2).^60^ The strategic cornerstone of their approach was the use of a biocatalytically hydroxylated enone building block (21), prepared by cytochrome P450-mediated oxidation of a sclareolide-derived enone, which served as the A/B-ring fragment and provided a critical functional handle for subsequent diversification. Retrosynthetically, (−)-azadiradione was traced to a common intermediate that could be accessed by convergent coupling of the enzymatic hydroxyenone fragment 21 (A/B-ring) with a highly substituted cyclopentenyl iodide fragment 30 (D/E-ring), followed by a hydrogen-atom-transfer (HAT)-mediated Giese cyclisation to close the central C-ring.

**Scheme 2:**
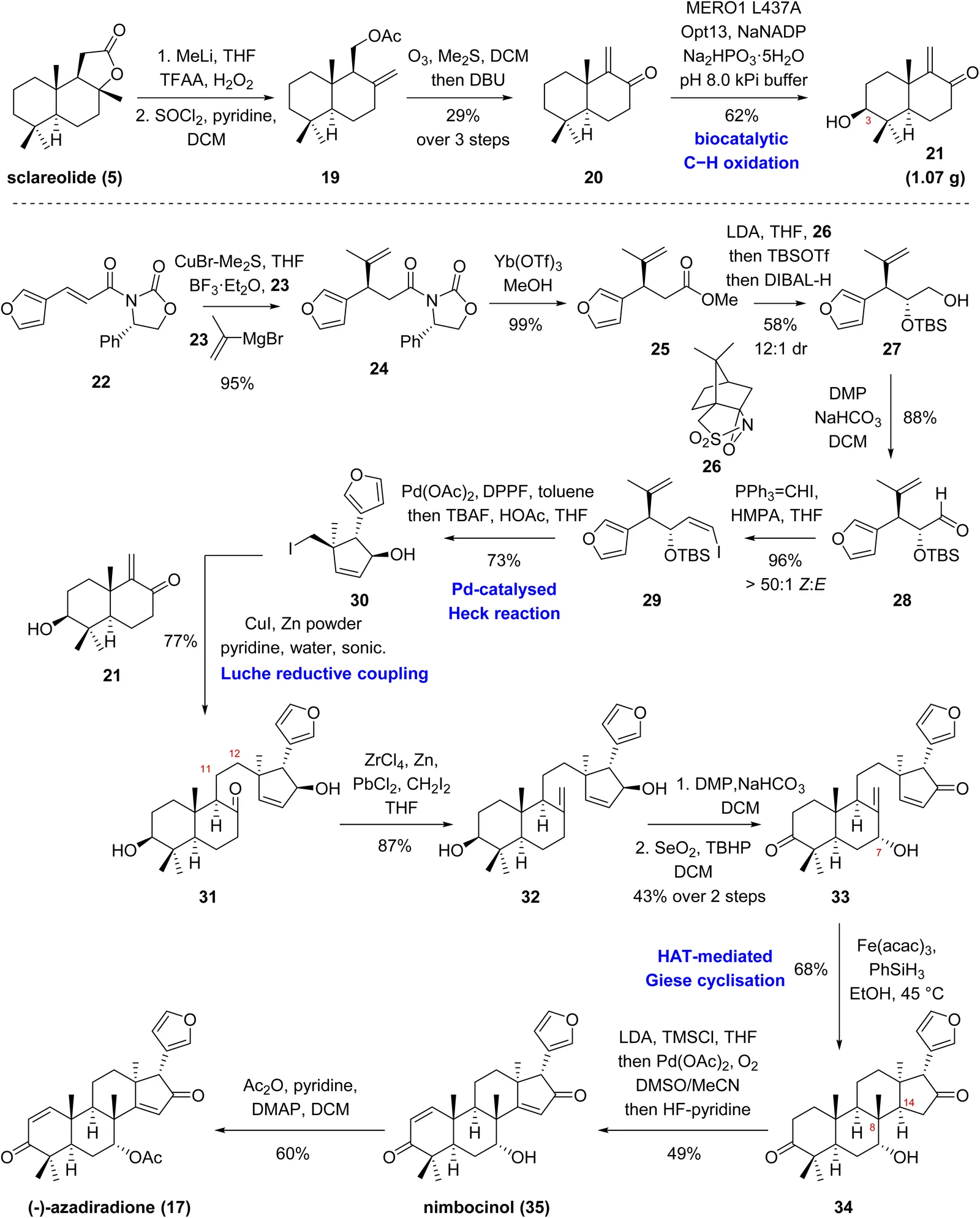
Renata's total synthesis of (−)-azadiradione.^60^

The synthesis commenced with the preparation of two fragments of comparable complexity. The A/B-ring fragment 21 was prepared from the inexpensive and abundant chiral pool starting material sclareolide (5).^61^ A five-step sequence involving MeLi addition, Baeyer–Villiger oxidation, dehydration of the C8 alcohol, ozonolysis, and acetate elimination furnished enone 20. Building on their previously developed biocatalytic platform for selective C3 oxidation of sclareolide derivatives,^56,62–64^ the authors screened a small panel of P450_BM3_ variants and identified the MERO1 L437A mutant as capable of oxidising 20 at its C3 carbon with minimal competing alkene epoxidation, providing the C3-hydroxylated fragment 21 on preparative scale (1.07 g obtained).^61^

Notably, the biocatalyst MERO1 L437A is an engineered variant of cytochrome P450_BM3_ from *Bacillus megaterium*.^61^ Unlike the non-heme α-ketoglutarate (α-KG)-dependent AndA dioxygenase (see Section 4.1.2), P450_BM3_ is a heme-containing monooxygenase that generates its active iron–oxo intermediate (Compound I) through NADPH-dependent electron transfer.^65^ Furthermore, MERO1 carries a fused P450-reductase domain on the same polypeptide, so no exogenous partner reductase needs to be reconstituted. NADPH itself, however, remains essential.^65,66^

For the D/E-ring fragment 30, copper-mediated Michael addition to the Evans auxiliary adduct 22 delivered 24 as a single diastereomer in 95% yield.^67^ Methanolysis of 24 furnished methyl ester 25,^68^ which was oxidised with Davis reagent to install the α-hydroxyl group. A telescoped sequence involving hydroxylation, TBS protection, and DIBAL reduction provided 27, and subsequent oxidation of the primary alcohol gave aldehyde 28. Stork-Zhao olefination with HMPA as additive afforded vinyl iodide 29 in exceptional yield and with greater than 50 : 1 *Z*/*E* selectivity.^69^ A Pd-mediated interrupted Heck reaction constructed the desired cyclopentene bearing three contiguous chiral centers,^70^ and *in situ* TBS deprotection with acidified TBAF delivered cyclopentenol 30.

With both fragments in hand, Luche reductive coupling of 21 and 30 by using activated zinc powder and CuI under sonication afforded coupled product 31.^71^ C8 methylenation of 31 was achieved by applying Jamison's modification of the Takai reaction,^72^ giving diol 32 in significantly improved yield over conventional Wittig conditions. Global oxidation of 32 with Dess–Martin periodinane (DMP) followed by SeO_2_-mediated Riley oxidation introduced the C7 allylic hydroxyl group in 33. The pivotal HAT-based Giese cyclisation established the intact limonoid carbon framework and provided 34.^73^ Saegusa desaturation of 34 with reduced Pd(OAc)_2_ loading and one-pot quenching with HF furnished nimbocinol (35). Finally, acetylation of 35 with acetic anhydride completed the synthesis of (−)-azadiradione (17) in 15 steps.

Moreover, they also accomplished the total synthesis of (+)-Corey's protolimonoid (37), the biogenetically prototypical 4,4,8-trimethyl-17-furanylsteroid framework originally designed by E. J. Corey.^74^ Selective MnO_2_ oxidation of the common diol intermediate 32 furnished ketone 36, which was then subjected to the Fe(acac)_3_/PhSiH_3_-mediated HAT-based Giese cyclisation employed in the (−)-azadiradione route above. This single step forged the C8–C14 bond, closed the central C-ring, and installed the C8 methyl group with the natural stereochemistry, completing (+)-Corey's protolimonoid (37) in only two further steps from 32 (Scheme 3).

**Scheme 3:**
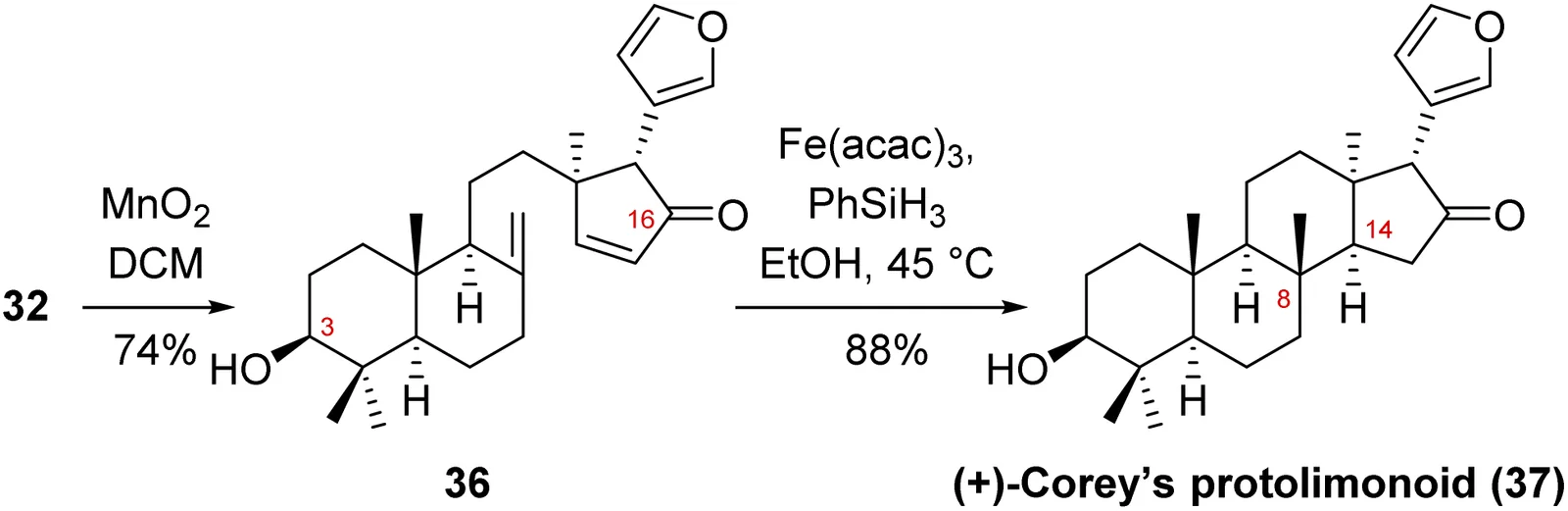
Renata's total synthesis of (+)-Corey's protolimonoid.^60^

### 3.3 Dethe's total synthesis of (+)-azadiradione and formal total synthesis of (−)-Corey's protolimonoid (2026)

Most recently, Dethe's group reported an enantioselective total synthesis of (+)-azadiradione (50) in 17 steps from a Hajos-Parrish ketone (HPK) derivative (Scheme 4).^75^ The defining methodological innovation of this work was the development of a novel sterically assisted hydroboration/boron chain-walking/oxidation protocol that enabled the highly regio- and stereoselective late-stage C7 hydroxylation, a transformation that had long been considered intractable in sterically congested polycyclic limonoid scaffolds.

**Scheme 4:**
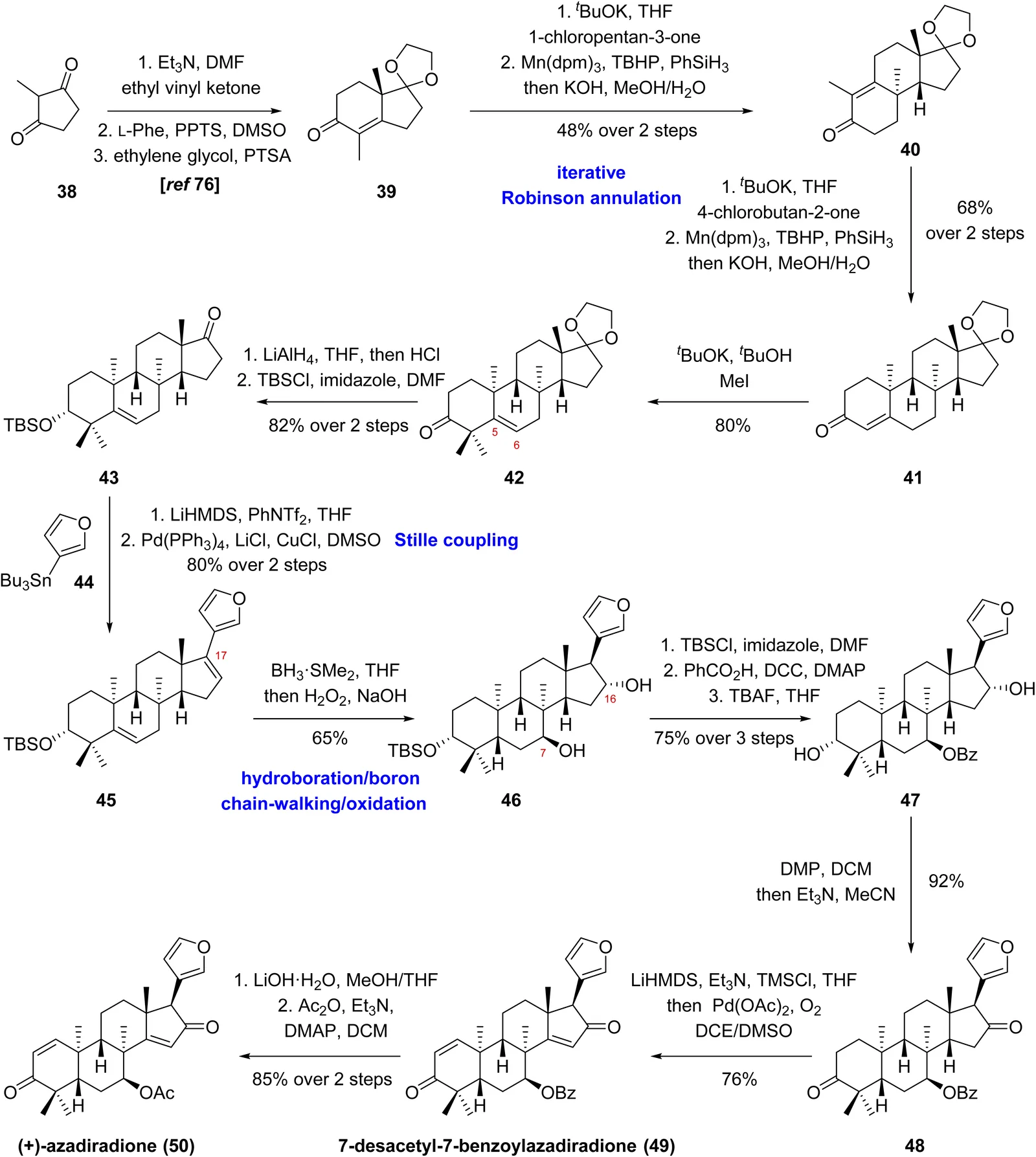
Dethe's total synthesis of (+)-azadiradione.^75^

Their synthetic route began with the preparation of (*S*)-enone 39, a HPK derivative, from 2-methylcyclopentane-1,3-dione 38 in three steps.^76^ The tetracyclic core was then rapidly assembled through an iterative Robinson annulation sequence. In the first iteration, alkylation of 39 with 1-chloropentan-3-one under ^*t*^BuOK was followed by a Mn(dpm)_3_/TBHP/PhSiH_3_-mediated HAT reaction (Shenvi's protocol)^77^ and KOH-promoted intramolecular aldol condensation, furnishing the tricyclic enone 40 in 48% yield over two steps. Subjecting 40 to the same sequence (alkylation/HAT/aldol) delivered the 6/6/6/5 tetracyclic enone 41 in 68% yield over two steps, thereby demonstrating the power of iterative annulation for efficient polycyclic framework construction.

With the tetracyclic core 41 in place, installation of the C5–C6 alkene *via*^*t*^BuOK/MeI methylation gave 42, and subsequent LiAlH_4_ reduction, ketal deprotection, and TBS protection afforded 43. The C17 furan moiety 44 was introduced through a Pd(PPh_3_)_4_/LiCl/CuCl-mediated Stille coupling with a furyl stannane reagent,^74^ providing intermediate 45. The pivotal dual hydroboration of 45 was then executed. The sterically encumbered C5–C6 alkene underwent the sterically assisted boron chain-walking/oxidation sequence to install the C7 α-hydroxyl, while the less hindered exocyclic alkene underwent conventional hydroboration–oxidation, yielding the diol 46 in 65% yield as a single diastereomer.^78^

Selective protection of the primary alcohol in 46 with TBSCl and benzoylation of the C7 hydroxyl with PhCO_2_H/DCC/DMAP, followed by global TBAF deprotection to give 47, and subsequent DMP/Et_3_N oxidation (carefully controlled to avoid C17 epimerization) afforded diketone 48. The endgame employed a Saegusa oxidation (LiHMDS/Et_3_N/TMSCl followed by Pd(OAc)_2_/O_2_) to introduce the α,β-unsaturation,^79^ delivering 7-desacetyl-7-benzoylazadiradione (49) in 76% yield. Finally, LiOH-mediated hydrolysis of the C7 benzoate and Ac_2_O/Et_3_N/DMAP acetylation provided (+)-azadiradione (50) in 85% yield over two steps.

In the same year, Dethe and co-workers further leveraged the iterative annulation logic for a concise formal synthesis of (−)-Corey's protolimonoid (55), starting from the same tricyclic enone 40 (Scheme 5).^80^ Application of the identical (alkylation/HAT/aldol) sequence furnished tetracyclic enone 51 in 70% yield over two steps. Installation of the A-ring gem-dimethyl group was achieved by reductive Birch methylation of 51 with lithium in liquid ammonia followed by quenching with iodomethane, affording tetracycle 52 in 85% yield as a single diastereomer. Reduction of the ketone in 52 and concomitant removal of the D-ring ketal were accomplished in a single step with LiAlH_4_ followed by acidic workup with HCl, giving alcohol 53 in 88% yield. Protection of the newly formed alcohol with TBSCl and imidazole furnished key intermediate 54 in 90% yield. The analytical data for 54 were in full agreement with those reported by Corey,^74^ culminating in the formal synthesis of (−)-Corey's protolimonoid (55) in seven steps from the HPK derivative (39).

**Scheme 5:**
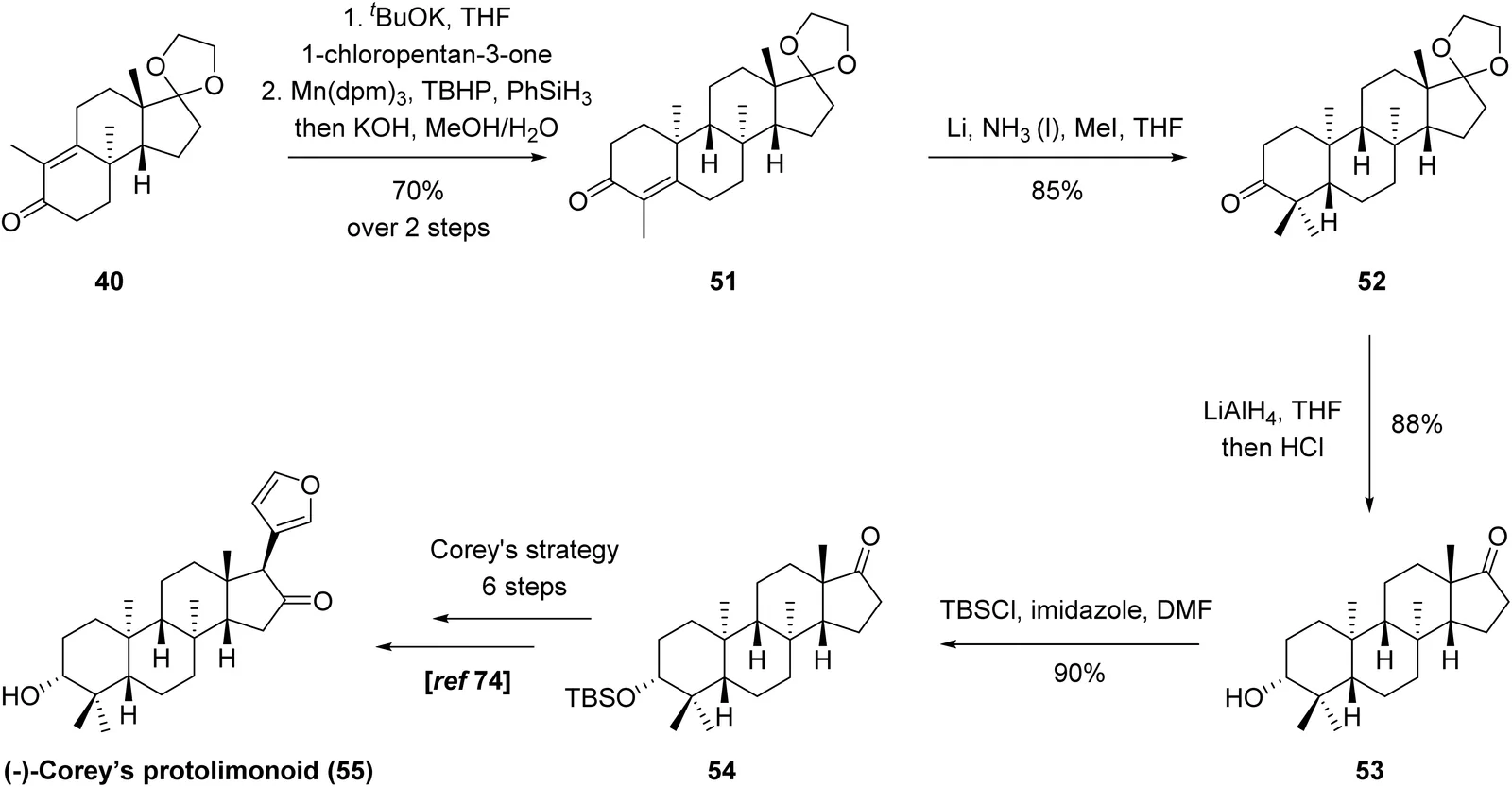
Dethe's formal total synthesis of (−)-Corey's protolimonoid.^80^

## 4. Total synthesis of ring-*seco* limonoids

### 4.1 Total synthesis of ring C-*seco* limonoids

#### 4.1.1. Qin and Yu's total synthesis of nimbolide (2023)

Nimbolide (69) is a prototypical ring C-*seco* limonoid isolated from the neem tree (*Azadirachta indica*).^81^ It features a densely functionalised hexacyclic framework containing an α,β-unsaturated ketone, a δ-lactone, a tetrahydrofuran C-ring arising from C12–C13 bond cleavage, and a C17 furan, together with multiple contiguous stereocenters including quaternary centers at C4, C8, and C10. Biologically, it acts as a covalent inhibitor of the PARylation-dependent E3 ligase RNF114,^82^ inducing supertrapping of PARylated PARP1 and PAR-dependent DNA-repair factors, which confers potent activity against *BRCA*-mutated cancers and overcomes resistance to conventional PARP inhibitors.^83^ Despite its structural and biological significance, efficient synthetic access to nimbolide remains challenging owing to its stereochemically complex and highly oxygenated scaffold.

In 2023, Qin and Yu's group reported the first total synthesis of nimbolide along with five related ring C-*seco* limonoid natural products and a variety of synthetic analogues, through a convergent pharmacophore-directed late-stage coupling strategy (Scheme 6).^84^ Their retrosynthetic analysis dissected the nimbolide scaffold into two advanced fragments of comparable complexity: an A/B-ring fragment embodying the essential enone–lactone pharmacophore (67) and a D/E-ring fragment incorporating the cyclopentenone and C17 furan (60). The critical tetrahydrofuran C-ring was to be forged through a sulfonyl hydrazone-mediated etherification followed by a radical cyclisation.

**Scheme 6:**
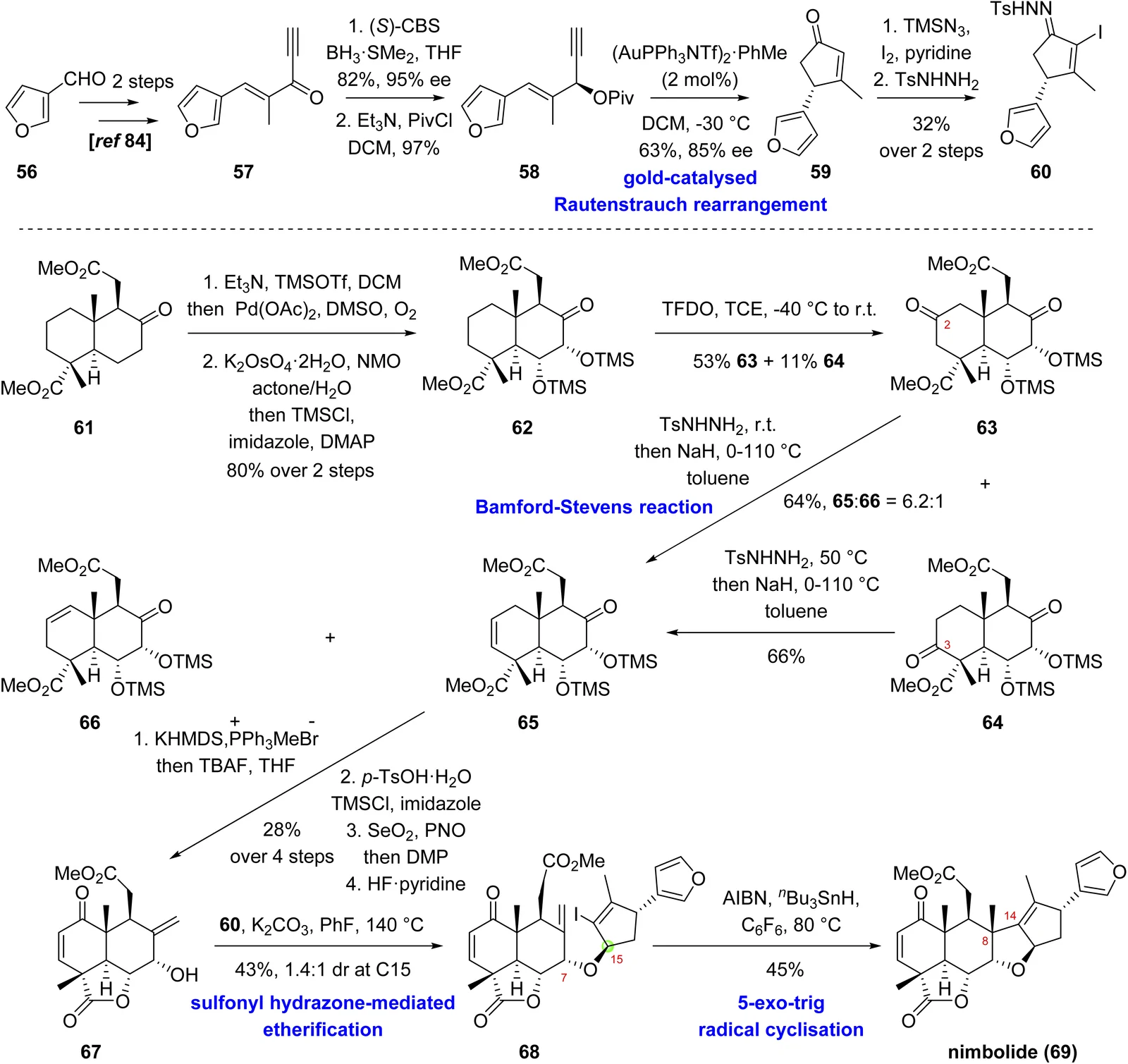
Qin and Yu's total synthesis of nimbolide.^84^

The synthesis commenced with the preparation of D/E-ring fragment (60). 3-Furaldehyde 56 was elaborated to alkyne-containing ketone 57. (*S*)-CBS reduction of 57 generated the chiral alcohol in 82% yield and 95% ee, and pivalate installation gave 58. The key gold-catalysed Rautenstrauch rearrangement of 58 then furnished chiral cyclopentenone 59 in 63% yield and 85% ee.^85^ α-Iodination with TMSN_3_ and I_2_ (ref. 86) followed by condensation with tosyl hydrazide afforded sulfonyl hydrazone 60 in 32% yield over two steps.

For the A/B-ring fragment (67), the known ketone building block 61 was prepared on a >350 g scale in three steps from abietic acid or five steps from dehydroabietic acid.^87^ Palladium-catalysed Saegusa oxidation^79^ of 61 followed by dihydroxylation and TMS protection afforded protected diol 62 as a single diastereomer on a decagram scale. The pivotal C–H oxidation at C2 was realised using methyl(trifluoromethyl)dioxirane (TFDO),^88^ delivering the desired C2 ketone 63 in 53% yield alongside the C3 ketone regioisomer 64 in 11% yield. Conversion of ketone 63 to the requisite olefin proved unexpectedly challenging under conventional dehydration conditions, but a one-pot Bamford–Stevens reaction furnished olefin 65 in moderate yield. A subsequent sequence of Wittig olefination, lactonisation, SeO_2_-mediated allylic oxidation,^89^ and TES deprotection provided A/B-ring fragment 67 in 28% yield over four steps, whose structure was unambiguously confirmed by single-crystal X-ray diffraction analysis.^84^

With both advanced fragments in hand, sulfonyl hydrazone-mediated etherification of 60 with 67 provided coupled products with a 1.4 : 1 diastereomeric ratio at C15 favouring the desired stereoisomer 68.^90^ The final radical cyclisation was initially problematic, as conventional AIBN/^*n*^Bu_3_SnH conditions in ordinary solvents afforded exclusively the undesired 6-*endo* product.^91^ The use of hexafluorobenzene as solvent proved critical,^92,93^ selectively promoting the desired 5-*exo*-trig radical cyclisation to complete the total synthesis of nimbolide (69). Additionally, the modularity of this strategy was further demonstrated by the divergent synthesis of numerous analogues with enhanced PARP1 trapping activity, as well as related natural products including 2,3-dihydronimbolide, 6-deacetylnimbin, nimbin, and nimbinene.^84^

#### 4.1.2. Li's formal total synthesis of nimbolide (2024)

In 2024, Li's group reported a chemoenzymatic formal synthesis of nimbolide (69) that showcased the power of engineered α-ketoglutarate (α-KG)-dependent dioxygenase AndA for installing the densely oxygenated A/B-ring oxidation pattern of labdane-type terpenoids (Scheme 7).^94^ It is worth framing this enzyme choice against the more familiar P450 monooxygenases used in Scheme 2. AndA is a non-heme Fe(ii)-dependent α-KG that couples substrate C–H hydroxylation to oxidative decarboxylation of α-KG into succinate and CO_2_, which was identified and structurally characterized by Abe's group.^95,96^

**Scheme 7:**
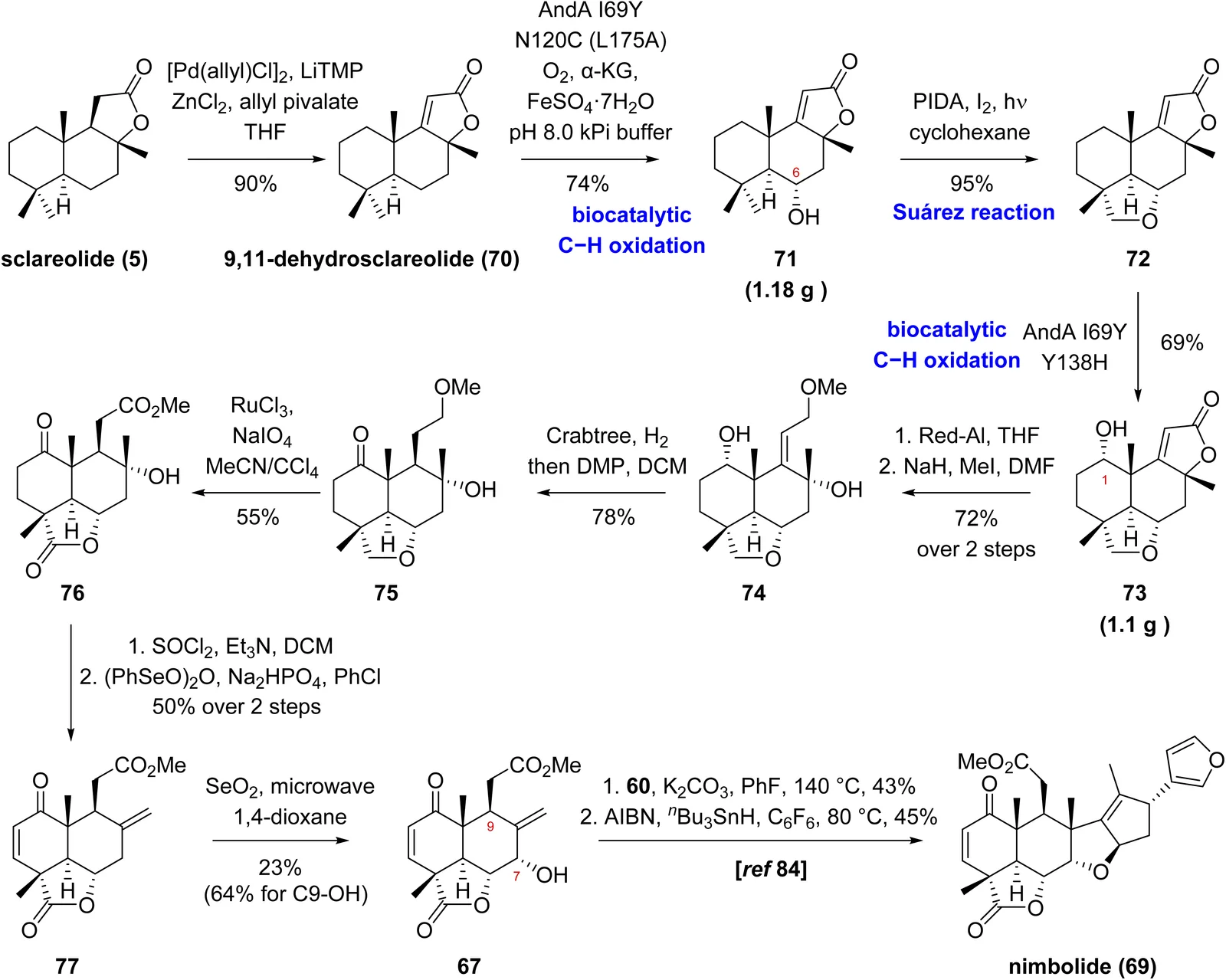
Li's formal total synthesis of nimbolide.^94^

AndA natively hydroxylates polyketide scaffolds and its comparatively open active site has made it an attractive starting point for directed evolution on terpene substrates.^96,97^ Operationally, AndA is particularly accessible to synthetic groups lacking dedicated P450 reconstitution facilities.^98^ Turnover requires only the purified enzyme, Fe(ii), α-KG, and ascorbate, with neither an NADPH-regeneration cycle nor a partner reductase.^99^

The authors first screened a small AndA library against six commercially available or readily preparable labdane-type substrates, ultimately selecting 9,11-dehydrosclareolide (70) as the optimal substrate. Inspection of the AndA crystal structure identified three bulky residues lining the active-site pocket (Ile69, Ile70, and Leu139); saturation mutagenesis at these positions was therefore pursued as a structure-guided rational strategy. The I69Y variant, which introduces a sterically demanding phenolic side chain that re-orients the bound substrate relative to the Fe(iv)-oxo centre, markedly improved conversion and delivered the C6α-hydroxylated product. Further rounds of saturation mutagenesis at positions 120 and 138 furnished variants that, respectively, retained C6α selectivity and switched regioselectivity to C1α-hydroxylation. Across this campaign, five contiguous C–H positions (C1, C2, C3, C5, and C6) on the labdane skeleton were selectively oxygenated (Fig. 3).^94^

**Fig. 3:**
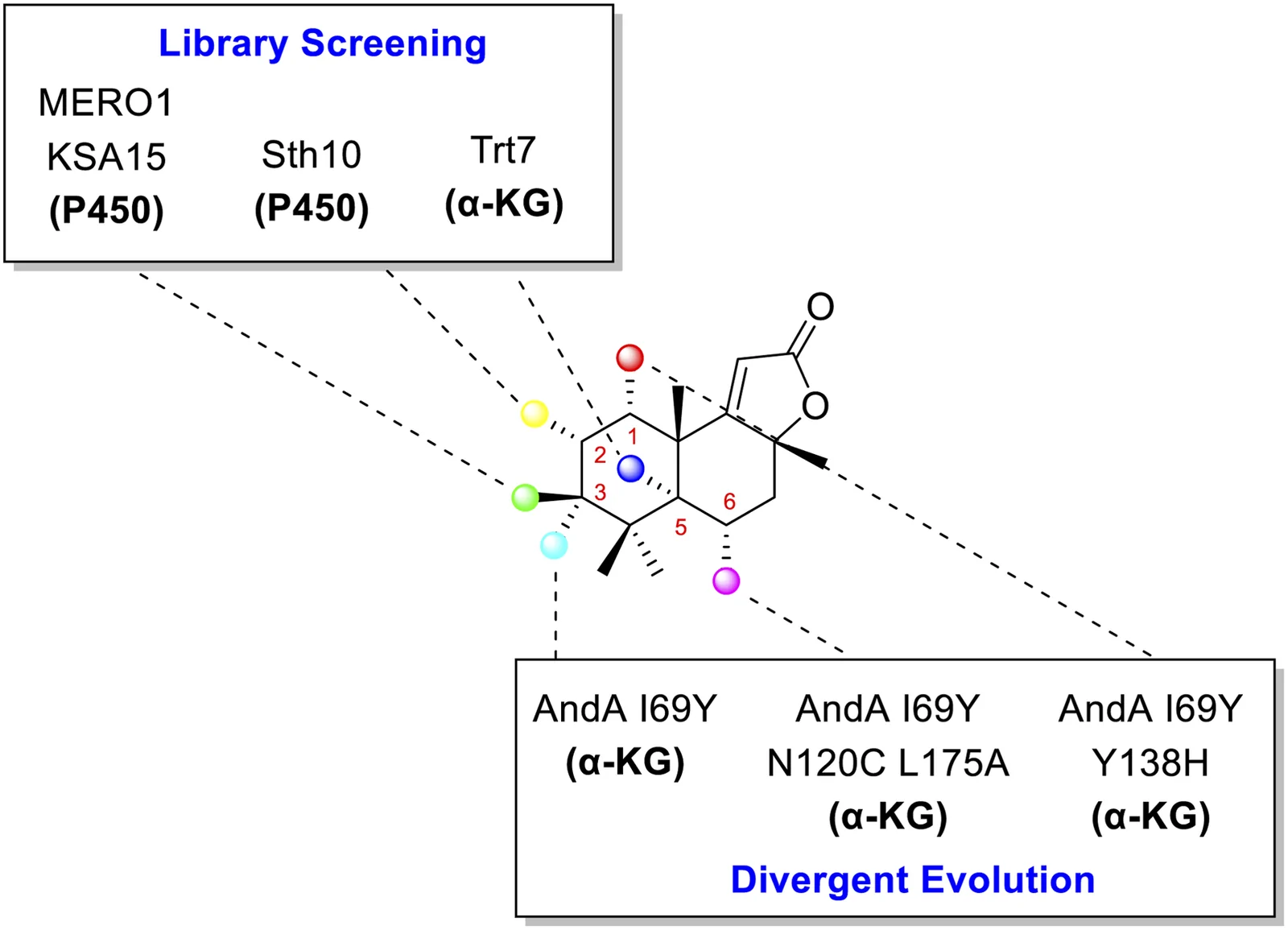
The enzymatic oxidation map of 9,11-dehydrosclareolide.^94^

Pairing these enzymatic oxidations with conventional chemistry gave twenty fully oxidized labdane derivatives, among which 73, bearing the nimbolide A/B-ring oxidation state, was accessed through a Suárez fragmentation (71 → 72)^100^ followed by AndA-variant C1α-hydroxylation (72 → 73). Further reduction of the lactone in 73 with Red-Al followed by *O*-methylation gave methyl ether 74 in 72% yield over two steps. Subsequently, hydrogenation of the endocyclic double bond by using Crabtree's catalyst and Dess–Martin oxidation afforded ketone 75 in 78% yield over two steps. The pivotal RuCl_3_/NaIO_4_ oxidation converted the two less hindered ether carbons to ester 76. Selective dehydration and subsequent oxidation with (PhSeO)_2_O/Na_2_HPO_4_ provided α,β-unsaturated ketone 77 in 50% yield over two steps.^101^ Finally, SeO_2_-mediated Riley oxidation under microwave irradiation delivered advanced intermediate 67 in 23% yield (64% for C9–OH), which is employed by Qin, Yu, and co-workers in their nimbolide synthesis,^84^ thus completing a protecting-group-free formal synthesis of nimbolide (69) in nine steps from 73 (thirteen steps from commercial sclareolide 5).

#### 4.1.3. Ma's total synthesis of nimbolide (2024)

Almost concurrently, Ma's group reported a concise, scalable, and protecting-group-free total synthesis of nimbolide (69) in only 12 steps (Scheme 8), which also served as a unified platform for the divergent synthesis of 52 ring C-*seco* limonoids spanning the nimbin-, salannin-, and nimbolinin-type classes.^102^ Their retrosynthetic strategy was centered on four key bond-forming reactions: a catalytic asymmetric intermolecular Diels–Alder reaction to forge the A-ring with the desired C4 and C5 stereochemistry, a diastereoselective Pd-catalysed reductive Heck reaction to construct the *trans*-fused A/B-ring system *via* C8–C9 bond formation, a sulfonyl hydrazone-mediated etherification, and a regioselective 5-*exo*-trig radical cyclisation to close the tetrahydrofuran C-ring.

**Scheme 8:**
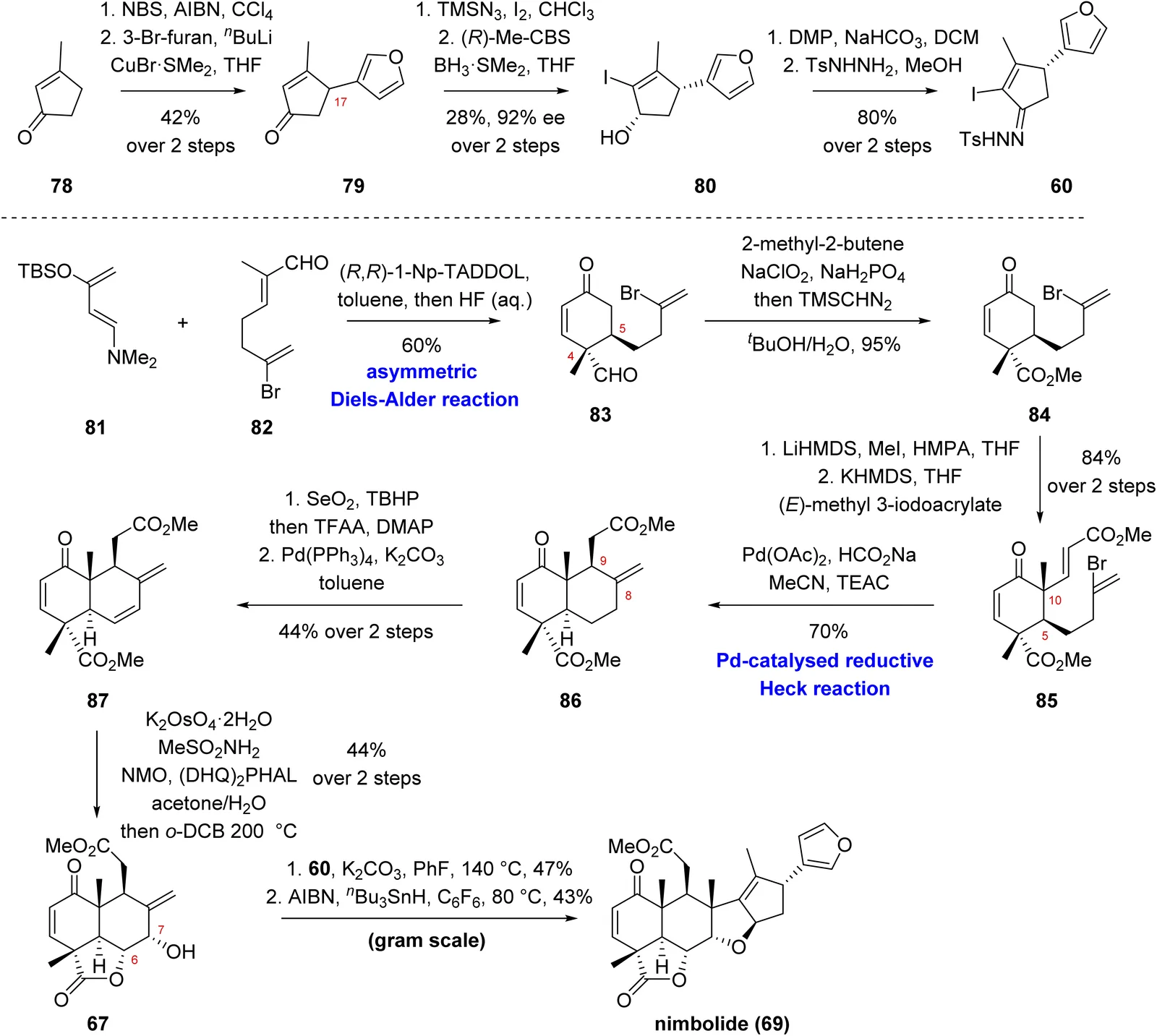
Ma's total synthesis of nimbolide.^102^

The D/E-ring fragment (60) was prepared from a commercially available enone 78*via* Wohl–Ziegler bromination and alkylation to install the C17 furan,^103^ followed by α-iodination,^86^ CBS reduction,^54^ DMP oxidation, and condensation with tosyl hydrazide. The preparation of A/B-ring fragment 67 began with an enantioselective intermolecular Diels–Alder reaction between Rawal's diene 81 and enal 82, catalysed by (*R*,*R*)-1-Np-TADDOL, which delivered the cycloadduct 83 in 60% yield and 72% ee after acidic workup. Pinnick oxidation and esterification (83 → 84) were followed by stereoselective methylation and Michael addition–elimination with (*E*)-methyl 3-iodoacrylate to install the C10 quaternary stereocenter in 85, with the pre-existing C5 stereocenter exerting sufficient steric bias to direct addition from the desired α-face. The initial attempt to close the B-ring *via* an intramolecular Rauhut–Currier reaction using PCy_3_ as the mediator afforded the cyclized product but with undesired C9 stereochemistry. This obstacle was circumvented by switching to a Pd-catalysed reductive Heck reaction of the corresponding vinyl bromide 85. After extensive screening of reaction parameters, the combination of HCO_2_Na as the reductant, TEAC as additive, and MeCN as solvent stereoselectively and efficiently furnished the *trans*-fused bicyclic product 86.

From the bicyclic product 86, site-selective allylic oxidation at C7 followed by esterification and Pd-mediated allylic elimination generated the diene intermediate 87.^104^ The subsequent chemo- and diastereoselective dihydroxylation of the C6–C7 double bond was achieved using K_2_OsO_4_ with (DHQ)_2_PHAL as ligand and NMO as oxidant, which not only improved the yield to 52% but also enriched the enantiomeric excess to 92% through kinetic resolution of the minor enantiomer.^105^ Thermal lactonisation at 200 °C in *o*-dichlorobenzene delivered the tricyclic A/B-ring fragment 67, whose structure was confirmed by X-ray crystallography.^102^

The convergent coupling of fragment 60 and 67*via* sulfonyl hydrazone-mediated etherification proceeded smoothly on a gram scale to afford the coupled product 68 in 47% yield, and subsequent AIBN/^*n*^Bu_3_SnH-mediated 5-*exo*-trig radical cyclisation completed the total synthesis of nimbolide (69).^84^ The versatility of this route was demonstrated by the divergent synthesis of 52 ring C-*seco* limonoids.^102^

#### 4.1.4. Sarlah's total synthesis of nimbolide (2025)

In 2025, Sarlah's group reported a stereoselective total synthesis of nimbolide (69) in a convergent 11-step sequence from α-methyl-(*R*)-carvone 92 (Scheme 9).^106^ The defining methodological innovation of this work was the development of a stereoselective palladium-catalysed borylative Heck cyclisation that simultaneously constructed the A-ring of the nimbolide core and installed a boronate ester at the C28 position, thereby streamlining access to the fully decorated *trans*-decalin moiety with oxidation at the quaternary C4 center.

**Scheme 9:**
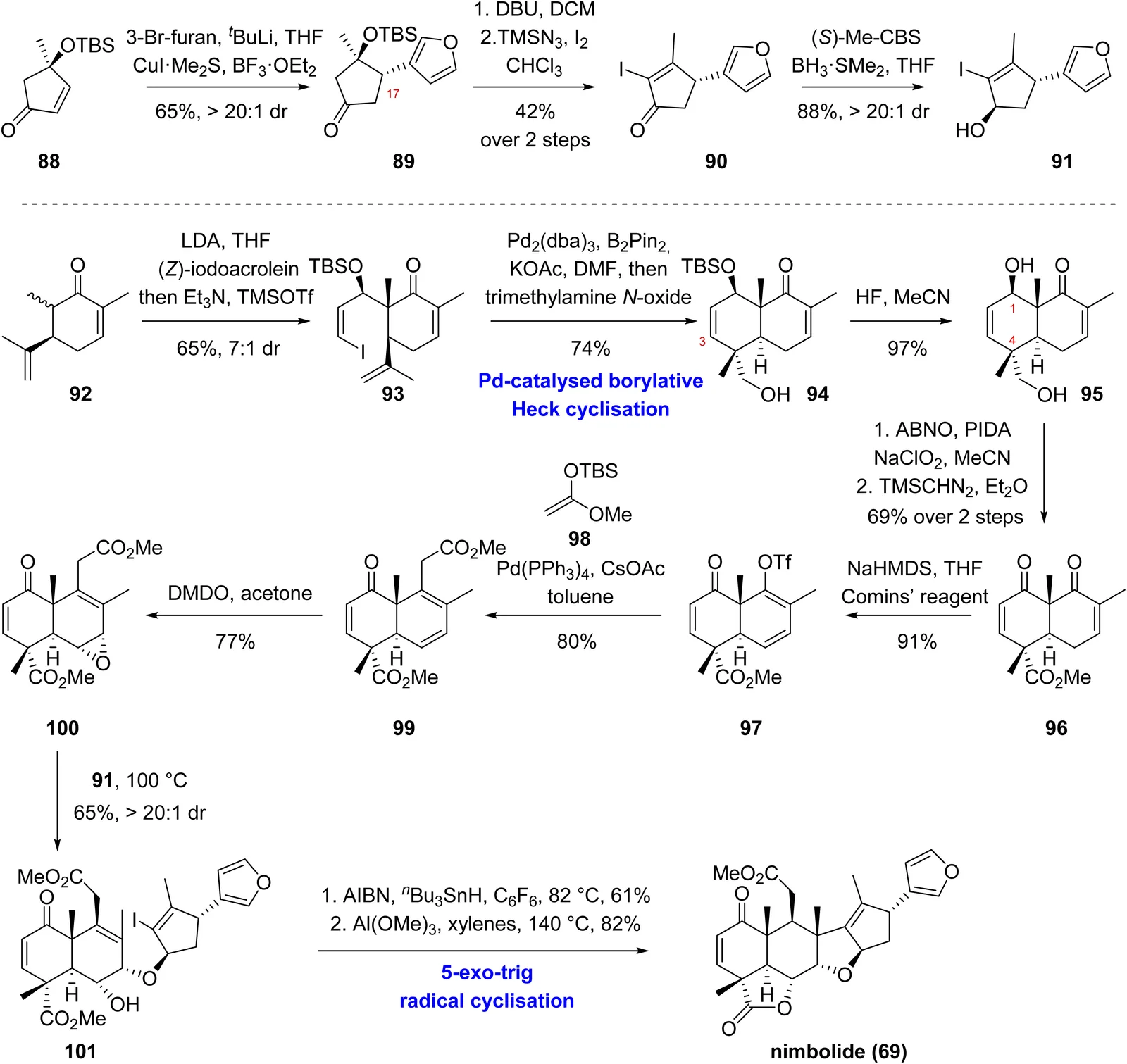
Sarlah's total synthesis of nimbolide.^106^

The nucleophilic alcohol fragment 91 was prepared separately in four steps from Maimone's cyclopentenone 88.^107^ Conjugate addition of a 3-furyl cuprate installed the C17 furan as a single diastereomer (89),^108^ β-elimination of the OTBS blocking group followed by α-iodination furnished iodoenone 90,^84^ and diastereoselective 1,2-reduction delivered alcohol 91.^109^ The construction of A/B-ring fragment commenced with an aldol reaction of α-methyl-(*R*)-carvone 92 with (*Z*)-iodoacrolein,^110^ followed by *in situ* TBS protection to afford vinyl iodide 93. The pivotal one-pot borylative Heck cyclisation was effected by sequential treatment of 93 with Pd_2_(dba)_3_, KOAc, and B_2_Pin_2_, followed by oxidation with trimethylamine *N*-oxide without intermediate workup,^111^ delivering the fully oxidized decalin 94 in 74% yield (11 : 1 dr in the crude mixture, single diastereomer after chromatography) and reproducibility on a decagram scale.

Removal of the TBS group in 94 with aqueous HF furnished diol 95, whose relative stereochemistry at C1 and C4 was unambiguously confirmed by X-ray diffraction. Concurrent oxidation of both alcohols using the sterically less demanding ABNO catalyst in place of TEMPO generated the A-ring enone and the C4 carboxylic acid,^112^ which was directly methylated without purification to provide dienone 96 in 69% yield over two steps. Subsequent deprotonation of the B-ring dienone followed by trapping with Comins' reagent afforded dienol triflate 97 in 91% yield.^113^ Palladium-catalysed cross-coupling of this sterically hindered triflate with a TBS silyl ketene acetal 98 (ref. 114) installed the methyl acetate side chain, delivering diene 99 with excellent reproducibility on a multigram scale. Regio- and diastereoselective epoxidation of the less hindered B-ring olefin from the bottom face with dimethyldioxirane (DMDO) then provided vinyl epoxide 100, the key electrophilic fragment for convergent coupling.

The critical fragment coupling proved uniquely challenging. Conventional Lewis or Brønsted acid-mediated epoxide openings afforded no reaction or poor diastereoselectivity, and the vinyl epoxide was prone to ionization and Meinwald rearrangement under acidic conditions.^115^ Ultimately, alcohol 91 and vinyl epoxide 100 were heated together neat, without solvent or acidic additives, in plastic reaction vessels to prevent acid-catalysed decomposition on glass surfaces.^116^ This afforded coupled product 101 in 65% yield as a single diastereomer. With the critical C–O bond forged, the endocyclic vinyl iodide in 101 underwent regioselective 5-*exo*-trig radical cyclisation under standard conditions to close the tetrahydrofuran ring in 61% yield. Notably, no competing 6-*endo* cyclisation was observed owing to the endocyclic nature of the olefin, a design feature that directly addressed the selectivity problem encountered in previous synthesis.^84,94,102^ Finally, late-stage lactonisation was carried out using aluminium methoxide as a mild Lewis acid in refluxing xylenes,^117^ conditions necessitated by the sensitivity of the intervening hydroxyester intermediate to both strong acid and base. This transformation completed the total synthesis of nimbolide (69) in 82% yield.

### 4.2 Total synthesis of ring D-*seco* limonoids

#### 4.2.1. Renata's total synthesis of gedunin (2022)

Gedunin (111) is a prototypical ring D-*seco* limonoid first isolated in 1960 by Taylor and co-workers from the West African timber *Entandrophragma angolense*.^118,119^ It exhibits a densely functionalised architecture characterised by a highly oxidized carbocyclic framework bearing an α,β-unsaturated ketone in the A-ring, a ring D-*seco* δ-lactone moiety, and a furyl substituent at C17, along with multiple contiguous quaternary stereocenters. Biologically, gedunin has attracted considerable attention as a heat shock protein 90 (HSP90) inhibitor that acts through inactivation of the co-chaperone p23,^120,121^ and it displays a broad spectrum of activities including antiproliferative, antimalarial, anti-inflammatory, and neuroprotective effects.^33^

In 2022, Renata's group achieved the first *de novo* total synthesis of gedunin in a concise 13-step longest linear sequence through a convergent chemoenzymatic strategy (Scheme 10).^61^ Two enabling features underpinned this work: the application of modern catalytic transformations to set the key quaternary centers in the carbocyclic core, and the use of a site- and chemoselective biocatalytic oxidation at C3 to establish a chemical handle for accessing the A-ring enone motif, a transformation that is particularly challenging chemically because stereoelectronic effects in the decalin framework direct conventional C–H oxidation to the undesired C2 position.

**Scheme 10:**
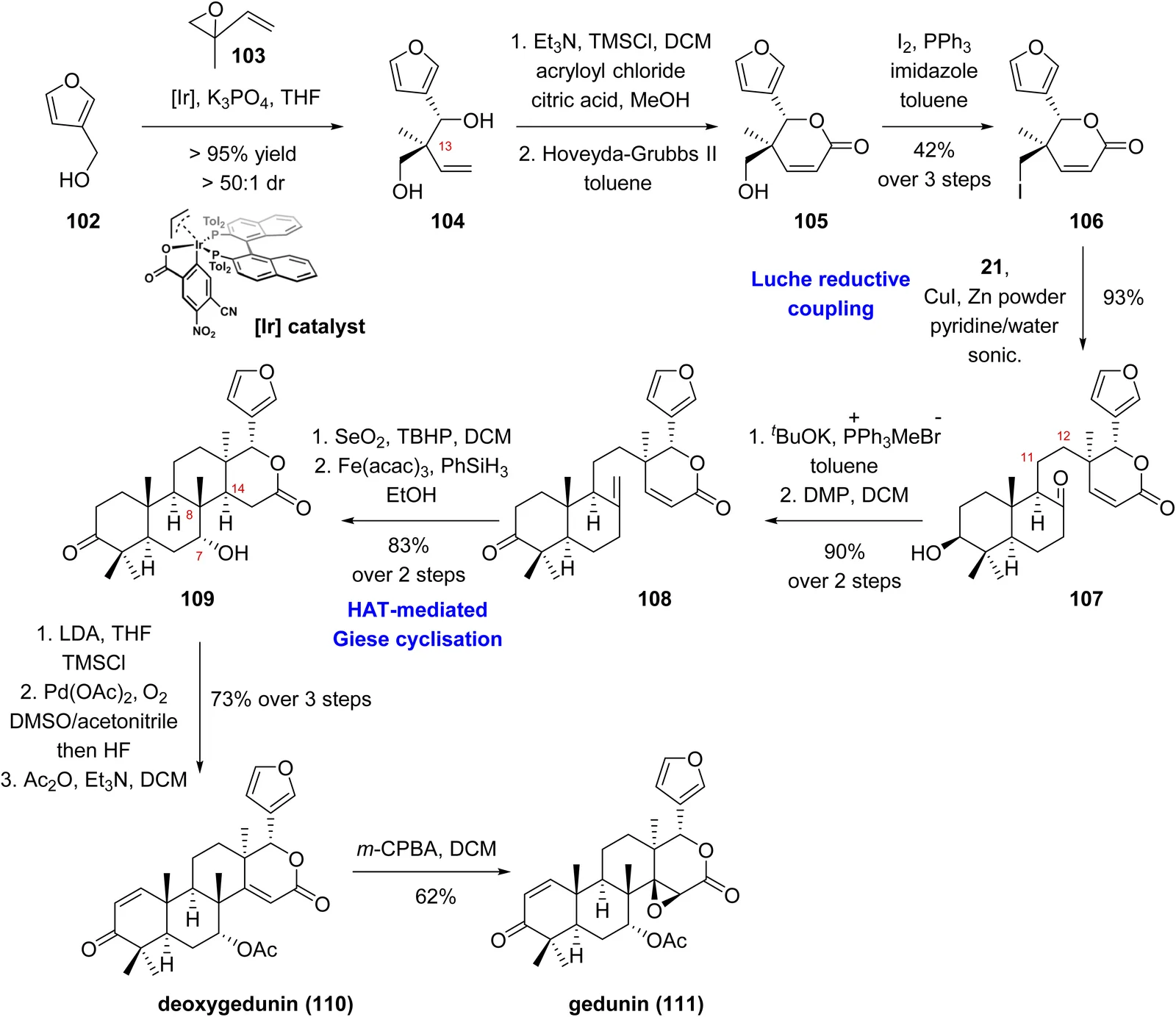
Renata's total synthesis of gedunin.^61^ This figure has been adapted from ref. 61 with permission from the American Chemical Society, copyright 2022.

The right-hand iodide fragment (106) was constructed through a catalytic enantioselective coupling. Furfuryl alcohol 102 was combined with isoprene monoxide 103 under Krische's hydrogen auto-transfer conditions,^122^ a redox-triggered C–C bond formation that installed the critical C13 all-carbon quaternary center with high diastereo- and enantioselectivity. Acrylation of the resulting secondary alcohol 104 followed by ring-closing metathesis furnished an unsaturated lactone 105, and routine conversion of the primary alcohol to the corresponding iodide completed fragment 106. In parallel, the left-hand enone fragment 21 was prepared from sclareolide through the five-step sequence and the P450_BM3_ MERO1 L437A hydroxylation described in Section 3.2.

With both fragments 21 and 106 in hand, union was achieved under Luche's conditions *via* a zincation/conjugate addition sequence.^71^ The coupled product 107 was then subjected to Wittig olefination to install the exocyclic methylene, followed by Dess–Martin oxidation of the C3 hydroxyl to reveal the A-ring ketone 108, while allylic oxidation at C7 introduced the necessary oxygenation at that position. A HAT-mediated intramolecular Giese cyclisation then forged the tetracyclic framework of 109 by closing the remaining carbocyclic ring,^73^ representing a strategic merger of biocatalytic oxidation with modern radical-based bond construction.

The final stages of the synthesis involved functional group manipulations to complete the oxidation pattern. Saegusa oxidation installed the A-ring α,β-unsaturated ketone, and acetylation at C7 delivered deoxygedunin (110). Finally, stereoselective epoxidation with *m*-CPBA converted deoxygedunin to gedunin (111), thus completing the first *de novo* total synthesis of this biologically important limonoid.

### 4.3 Total synthesis of ring A,D-*seco* limonoids

#### 4.3.1. Gui's total synthesis of deoxylimonin (2026)

Deoxylimonin (129), a prototypical ring A,D-*seco* limonoid, was first isolated from grapefruit (*Citrus paradisi*) seeds and has subsequently been identified in various species of the Rutaceae family.^123^ It possesses a unique 6/5/6/6/6-pentacyclic framework characterised by a ring A,D-*seco* skeleton bearing a fused tetrahydrofuran/δ-lactone motif, a 3-furanyl lactone moiety at C17, and multiple contiguous all-carbon quaternary stereocenters. Unlike limonin, which bears an epoxide at C14–C15, deoxylimonin lacks this oxygenation, rendering it a strategically important target for establishing synthetic access to the broader ring A,D-*seco* limonoid family. In 2015, Yamashita's group accomplished the first total synthesis of limonin in 35 linear steps.^124^

In 2026, Gui and co-workers reported a concise total synthesis of deoxylimonin (129) based on a bioinspired skeletal reorganization strategy (Scheme 11).^125^ The route hinges on four key transformations, namely a stereoconvergent radical polyene cyclisation to assemble the 6/6/6 tricyclic system, an oxime-directed Baldwin-Sanford oxidation to hydroxylate the unactivated C7–H bond, a titanium-mediated intermolecular aldol reaction to install the 3-furanyl lactone moiety with two contiguous stereogenic centers, and a biomimetic transesterification/oxa-Michael addition (TOMA) cascade to construct the intricate tetrahydrofuran/δ-lactone-fused motif.

**Scheme 11:**
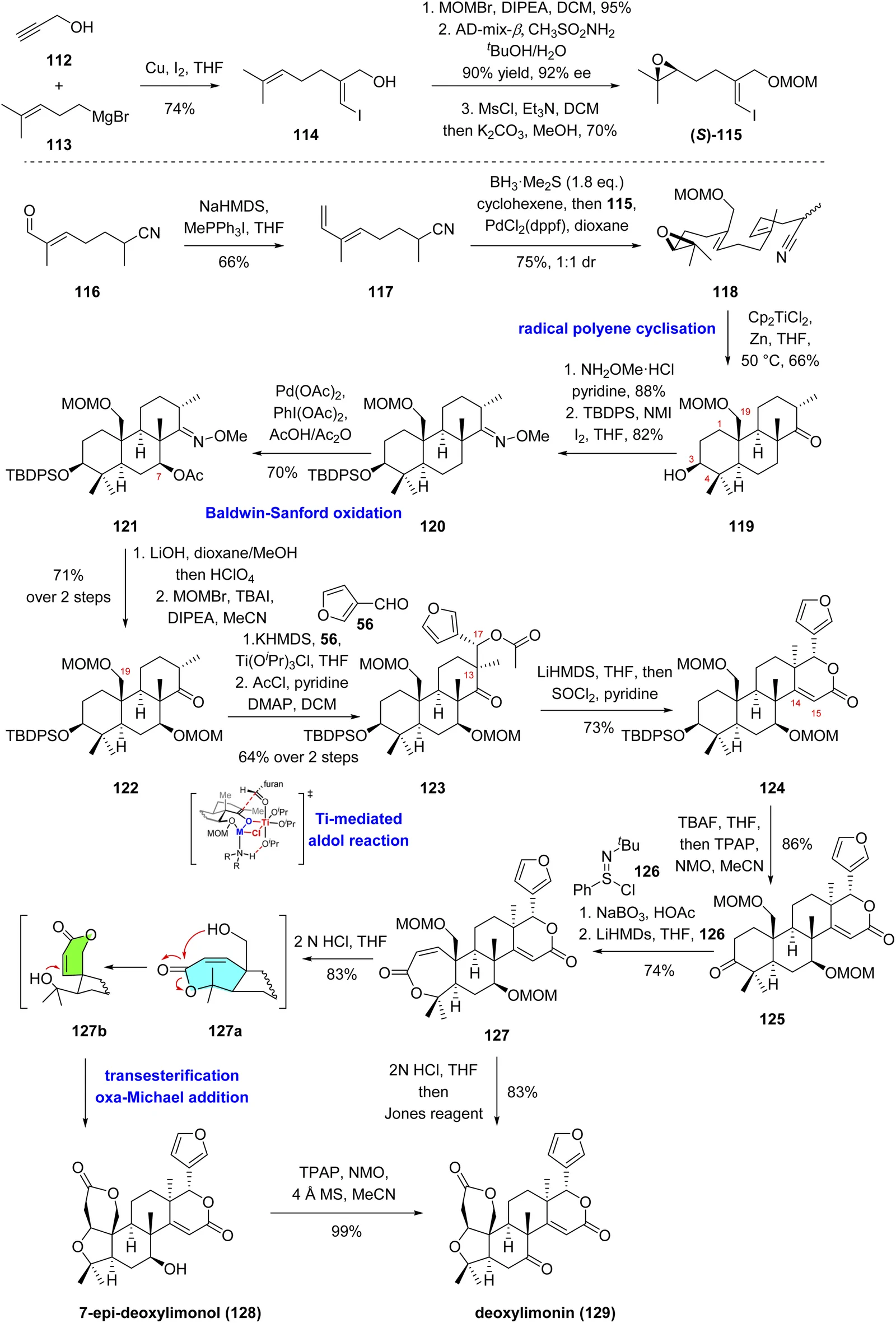
Gui's total synthesis of deoxylimonin.^125^ This figure has been adapted from ref. 125 with permission from the American Chemical Society, copyright 2026.

The synthesis commenced with the stereocontrolled preparation of the tricyclic ketone core. CuI-catalysed Grignard addition of 113 to propargyl alcohol 112, followed by quenching with I_2_, furnished vinyl iodide 114 in 74% yield as a single (*Z*)-stereoisomer.^126^ MOM protection of 114 and subsequent Sharpless asymmetric dihydroxylation delivered diol (90% yield, 92% ee),^127^ which was elaborated to enantiomerically enriched epoxide (*S*)-115.

The preparation of the radical polyene cyclisation substrate 118 began with Wittig olefination of known aldehyde 116 to deliver diene 117,^128^ which underwent regioselective hydroboration with dicyclohexylborane followed by Suzuki–Miyaura coupling with (*S*)-115, leading to epoxynitrile 118 as a 1 : 1 mixture of diastereomers. Gratifyingly, radical polyene cyclisation of 118 at 50 °C afforded tricyclic ketone 119 in 66% yield as a single diastereomer.^129^

With the tricyclic framework in hand, attention turned to the challenging site-selective C7 hydroxylation. Treatment of ketone 119 with methoxyamine produced the desired (*E*)-oxime as a single stereoisomer, which was followed by iodine-promoted silylation with *tert*-butyldiphenylsilyl chloride to afford 120.^130^ The unactivated equatorial C7–H bond was then hydroxylated *via* an oxime-directed Baldwin-Sanford oxidation using Pd(OAc)_2_ and PhI(OAc)_2_, furnishing C7β-acetate 121 as a single diastereomer.^131^ Conversion of 121 to ketone 122 was achieved through a two-step sequence involving acetate and oxime hydrolysis, followed by protection of the C7 and C19 hydroxyl groups as MOM ethers.

The installation of the 3-furanyl lactone moiety through an intermolecular aldol reaction between ketone 122 and furan 56 was particularly challenging, as it required the simultaneous establishment of the contiguous C13 quaternary and C17 stereocenters. Initial attempts with lithium diisopropylamide or Mukaiyama aldol conditions gave no reaction, and conventional titanium enolate formation was likewise unproductive. The key to success lay in the addition sequence. Addition of the lithium enolate of 122, generated with KHMDS as the optimal base, to a solution of Ti(O^*i*^Pr)_3_Cl and furan 56 delivered exclusively the *threo* aldol product in 50% yield.^132^ Reducing the KHMDS loading to 3 equivalents further improved the yield to 77%. Notably, this aldol product could be quantitatively converted back to 122 under basic conditions, indicating that suppression of the retro-aldol pathway by the titanium reagent and precise control of the addition sequence were essential for the success of this reaction.

Completion of the synthesis from aldol adduct involved acetylation to generate 123, followed by an intramolecular aldol reaction to produce a C14α-alcohol intermediate, which was treated *in situ* with SOCl_2_ to afford lactone 124.^133^ Removal of the C3 silyl group and subsequent Ley-Griffith oxidation delivered 125, which underwent Baeyer–Villiger oxidation with NaBO_3_/HOAc followed by Mukaiyama dehydrogenation to furnish unsaturated lactone 127.^134^ Treatment of 127 with 2 N HCl smoothly triggered removal of the two MOM protecting groups and a concomitant biomimetic TOMA cascade (127a → 127b),^135^ delivering 7-*epi*-deoxylimonol (128) in 83% yield. Final oxidation of 128 completed the synthesis of deoxylimonin (129), which could also be obtained directly from 127*via* a one-pot procedure (2 N HCl followed by Jones reagent, 83% overall).

#### 4.3.2. Newhouse's total synthesis of deoxylimonin (2026)

In the same year, Newhouse's group disclosed a *de novo* total synthesis of deoxylimonin (129) through a strategy that integrated multiple local computational models into the retrosynthetic analysis (Scheme 12).^136^ The strategic cornerstone was assembly of a key carbocyclic tricycle in only eight synthetic steps, followed by a modern ring-cleavage/ring-formation sequence using a late-stage oxa-Michael cyclisation. What distinguishes this work from the other syntheses is that two high-risk bond-forming steps were interrogated by dedicated local DFT models before synthetic chemistry was committed.

**Scheme 12:**
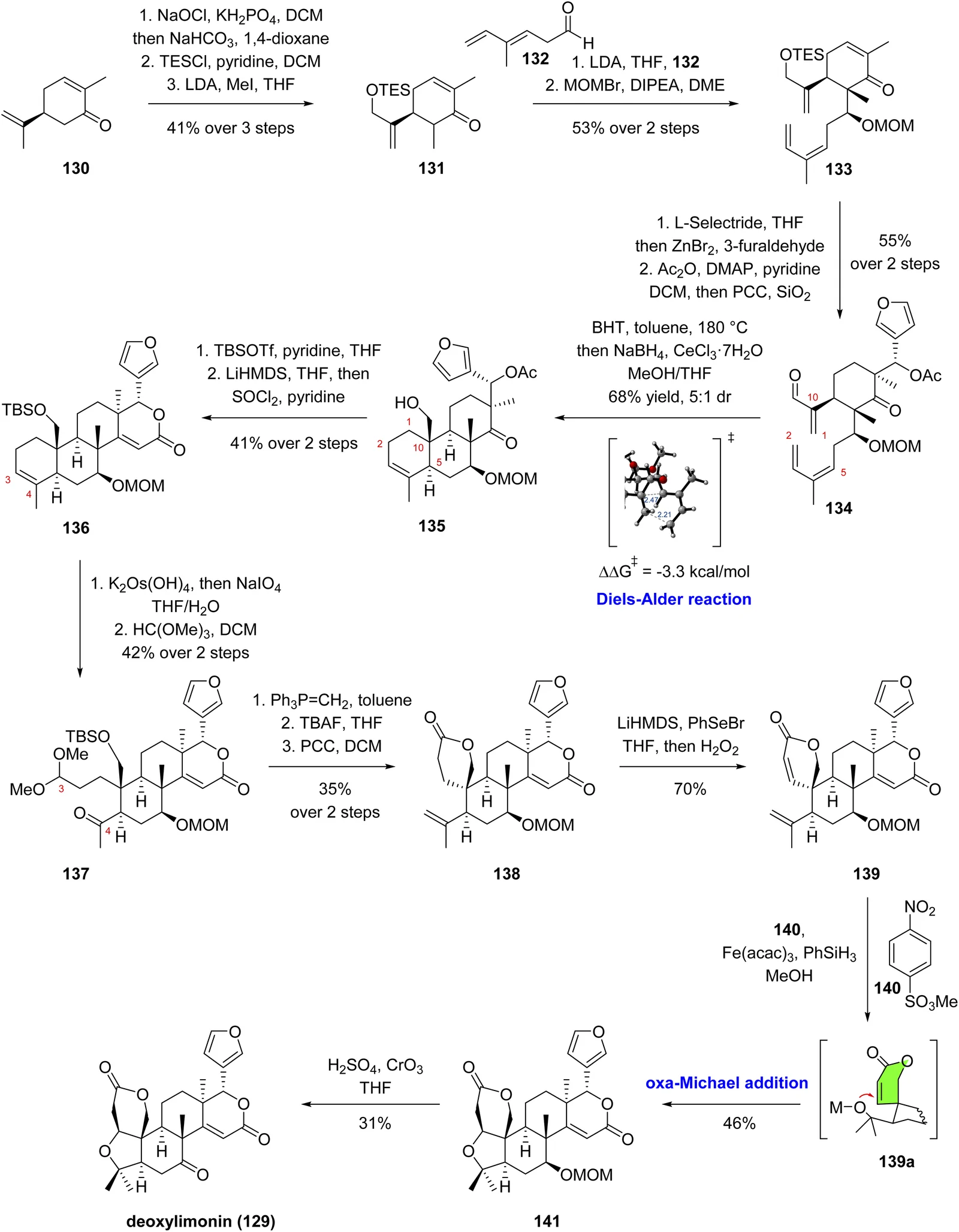
Newhouse's total synthesis of deoxylimonin.^136^ This figure has been adapted from ref. 136 with permission from the American Chemical Society, copyright 2026.

The first model addressed the thermodynamic feasibility of the closing oxa-Michael step. Because oxa-Michael cyclisations are intrinsically reversible and compete with the open lactone form, B3LYP-D3/6-31++G(2d,p)//B3LYP-D3/6-31G(2d,p) calculations were used to screen forty candidate B-ring substituents R and compare the free energy of the open precursor against the cyclised adduct. The closed form was thermodynamically favoured across all forty substrates (median Δ*G*_avg_ = −9.6 kcal mol^−1^), identifying the oxa-Michael disconnection as viable and guiding the functional-group selection carried through the route. The second model addressed the stereochemical outcome of the intramolecular Diels–Alder reaction that assembles the tricyclic core. M06-2X/6-311+G(d,p)//M06-2X/6-31G(d) transition-state calculations predicted the *endo* pathway to be favoured over *exo* by ΔΔ*G*^‡^ = −3.3 kcal mol^−1^, and the experiment subsequently delivered the *endo* adduct as a 5 : 1 diastereomeric mixture, consistent with the DFT-predicted *endo* selectivity.^136^

The synthesis began with functionalisation of the commercially available chiral pool starting material (*S*)-(+)-carvone (130). A sequence of allylic chlorination with NaOCl and displacement under basic aqueous conditions furnished the necessary allylic alcohol, which was protected with TESCl to afford the protected intermediate.^137^ Subsequent methylation provided the methylated enone 131 in 41% yield over three steps. An aldol reaction with aldehyde 132 proceeded smoothly to give the desired alcohol on a decagram scale,^138^ which was subsequently protected as a MOM ether to form intermediate 133 in 53% yield over two steps. Selective conjugate reduction of α,β-unsaturated ketone 133 with L-Selectride was followed by a direct aldol reaction with 3-furaldehyde in the presence of ZnBr_2_, installing the crucial furanyl moiety in 70% yield on a decagram scale. A one-pot protocol was then devised to acetylate the resulting alcohol and oxidise the silyl ether to the corresponding enal. After DMAP-catalysed acetylation with acetic anhydride, PCC oxidation in the presence of silica facilitated both deprotection of the silyl ether and oxidation of the resulting allylic alcohol, affording enal product 134 in 55% yield over two steps on a decagram scale.

The intramolecular Diels–Alder reaction of 134 then delivered the *endo* cycloadduct as the major diastereomer in a 5 : 1 ratio,^139^ matching the *endo*/*exo* selectivity predicted by the transition-state calculations discussed above. Lowering the reaction temperature greatly reduced the rate, whereas prolonged heating eroded diastereoselectivity. The crude product was carried directly into a Luche reduction to furnish alcohol 135 in 68% yield on a multigram scale.^140^ The carbocyclic core 135 was thus assembled in only eight steps and serves as a divergent point for accessing other ring D-*seco* limonoids.

The following sequence of transformations facilitated formation of the full tetracyclic skeleton. Alcohol 135 was quantitatively protected as a silyl ether using TBSOTf, and the resulting tricyclic ketone underwent intramolecular cyclisation upon treatment with LiHMDS to generate an intermediate bearing a tertiary alcohol, which was eliminated in the same pot with thionyl chloride to afford desired intermediate 136 with the D-ring intact in 41% yield over the two steps. Efforts to achieve direct condensation using Burgess reagent or Martin's sulfurane were unsuccessful, and some decomposition was observed upon treatment with thionyl chloride, possibly attributable to its inherent acidity.^141^

Access to intermediate 136 enabled the key ring cleavage and formation sequence. Regioselective Os-catalysed dihydroxylation followed by oxidative cleavage with NaIO_4_ provided a dicarbonyl compound, which was selectively protected to give dimethyl acetal 137. A three-step sequence of Wittig olefination followed by acidic workup,^142^ TBAF deprotection, and PCC oxidation of the ensuing lactol constructed the spirocyclic lactone A-ring 138 in 35% yield over three steps. Following one-pot selenylation and oxidation, the desired α,β-unsaturated lactone 139 was obtained in 70% yield, setting the stage for hydration and the oxa-Michael reaction.

Initial attempts at oxymercuration, acid-catalysed hydration, or typical metal-hydrogen-atom-transfer (MHAT) conditions did not yield hydration products, necessitating an approach using a milder oxidant. Gratifyingly, Studer's recently developed anaerobic Mukaiyama-type hydration utilising nitroarenes 140 as oxidants proved effective,^143^ and the resulting hydrated intermediate 139a directly underwent the oxa-Michael reaction to complete construction of the tetrahydrofuran/δ-lactone fused rings in intermediate 141. Although complex mechanistic studies would be required to differentiate between radical and ionic pathways, the computational studies conducted during the design stages are applicable to both mechanistic possibilities.^144^ Finally, a one-pot sequence of deprotection and oxidation led to the formation of the natural product deoxylimonin (129). The modest yield of this final step was attributed to unidentified byproducts that lacked furan functionality.

### 4.4 Total synthesis of ring B,D-*seco* limonoids

#### 4.4.1. Newhouse's total synthesis of (±)-andirolide N (2017)

Andirolide N (157) is a ring B,D-*seco* limonoid isolated from the flowers of the Amazonian mahogany tree (*Carapa guianensis*) by Tanaka and co-workers in 2012.^145^ It possesses a synthetically demanding bicyclo[3.3.1]nonane ring system with a bridging tetrahydrofuran ring appended, representing one of the most architecturally complex members of the limonoid family. Like many limonoids, andirolide N exhibits a broad spectrum of biological activities including antimalarial, anticancer, and anti-inflammatory properties.^20^

In 2017, Newhouse's group reported the total synthesis of (±)-andirolide N in a 12-step sequence from commercially available dihydrocarvone (Scheme 13).^146^ The strategy centered on using the degraded limonoid iso-odoratin as a platform for divergent assembly of the limonoid architecture, with the A-ring, a site of considerable structural diversity in this family, introduced at a late stage. The synthesis also provided experimental support for the biosynthetic proposal that andirolide N arises from 8α-hydroxycarapin (156) through acid-mediated reorganization.^146^

**Scheme 13:**
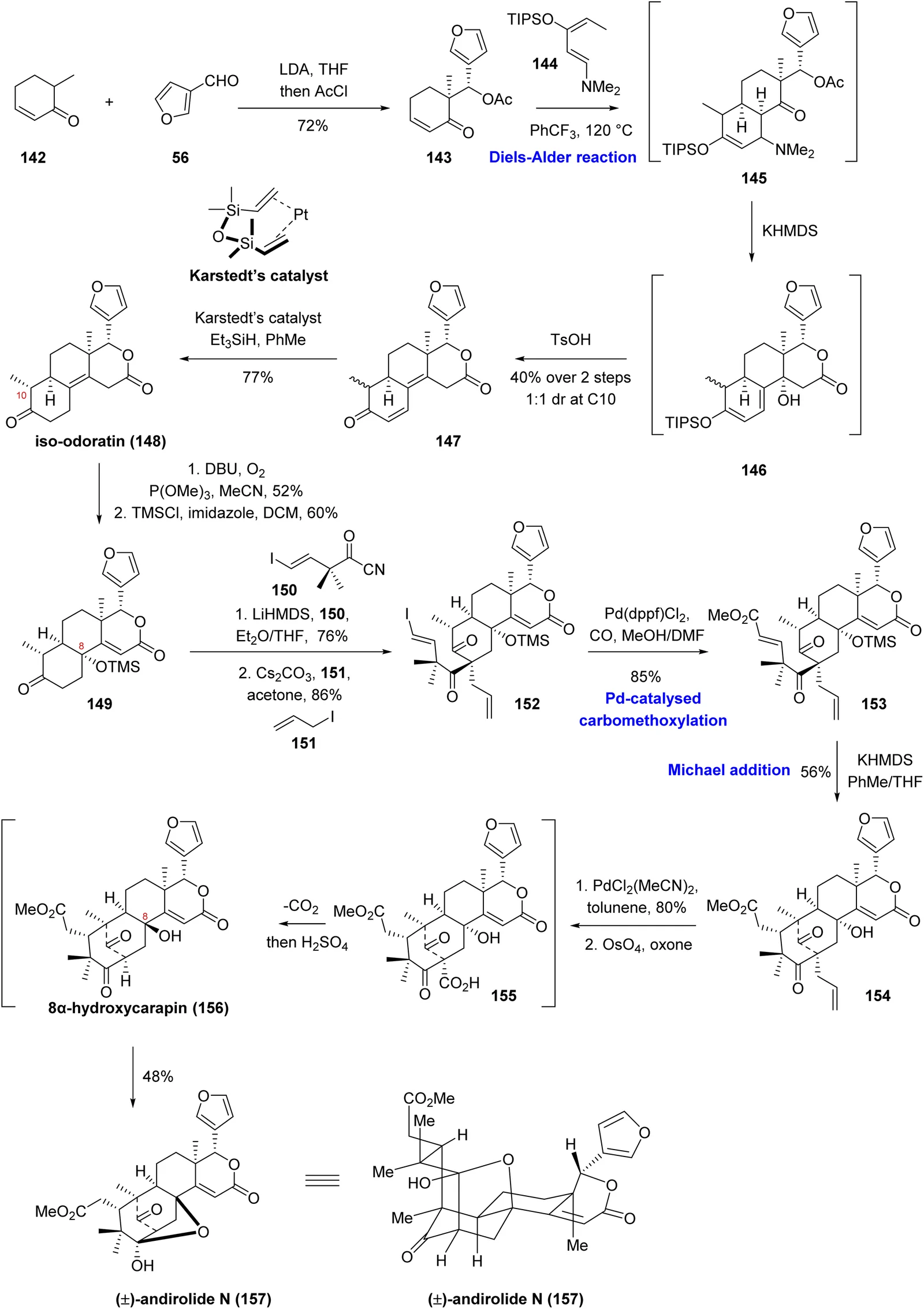
Newhouse's total synthesis of (±)-andirolide N.^146^

The synthesis commenced with the preparation of 6-methylcyclohexenone 142 from commercially available dihydrocarvone in one step.^147^ A diastereoselective aldol reaction between the lithium enolate of 142 and 3-furaldehyde 56, followed by quenching the intermediate alkoxide with acetyl chloride, furnished acetate 143 in 72% yield. Thermal Diels–Alder reaction of 143 with a Rawal-type diene 144 afforded the cycloadduct 145 as an uncharacterized mixture of diastereomers. Treatment of 145 with KHMDS effected an intramolecular acetate aldol reaction followed by β-elimination of the dimethylamino group, and subsequent exposure of the crude product 146 to *p*-toluenesulfonic acid triggered desilylation and dehydration to deliver 147 in 40% overall yield over two steps as a 1 : 1 mixture of diastereomers at C10.

With the carbon framework of iso-odoratin in place, the reduction of the doubly vinylogous 1,7-dicarbonyl compound 147 proved to be uniquely challenging, as commonly employed enone reduction conditions preferentially deprotonated this acidic substrate rather than effecting alkene reduction. The formation of the extended enolate of 147 was confirmed by quenching purple reaction mixtures with deuterated solvent, resulting in loss of colour and incorporation of deuterium. After extensive investigation, Karstedt's catalyst (tris(tetramethyldivinyldisiloxane)diplatinum) with triethylsilane was found to cleanly reduce 147,^148^ affording iso-odoratin (148) as a single diastereomer in 77% yield after acidic aqueous workup. The use of triethylsilane proved more efficient than other silanes (TIPSiH, DMPSiH, TMDS, or PMHS), correlating with previously observed differences in rates of hydrosilylation. The four-step sequence from 6-methylcyclohexenone 142 to iso-odoratin (148) proceeded in 22% overall yield and could be conducted on a decagram scale.

The regio- and stereoselective hydroxylation at C8 of the limonoid framework is a well-established challenge in limonoid semisynthesis,^149^ as oxidation of odoratin is known to lead to aromatisation of the B-ring. Guided by this precedent, the authors employed iso-odoratin (148) as an alternative substrate and discovered that direct treatment of the crude hydrosilylation mixture containing 148 with oxygen, trimethyl phosphite, and DBU effected a formal alkene hydration to install the C8 hydroxyl group as a single regio- and diastereomer.

Construction of the bicyclo[3.3.1]nonane ring system was initiated by acylation of the lithium enolate derived from 149 with acyl cyanide 150, furnishing 1,3-diketone in 76% yield. The use of an acylation strategy rather than an aldol/oxidation approach allowed for redox-neutral installation of the 1,3-dicarbonyl functionality while precluding retro-aldol and aldol condensation side reactions.^150^ Before the intramolecular Michael addition could be performed, the α-position of the 1,3-diketone required blocking to enable deprotonation at the γ-position (C10). After extensive screening of conventional blocking groups (–OR, –SR, –X),^151^ a carbon-based allyl group was found to be optimal. Thus, α-allylation of 1,3-diketone with allyl iodide 151 and caesium carbonate proceeded from the less hindered convex face to afford 152 as a single diastereomer in 86% yield, enforcing the necessary geometry for the subsequent bicyclo[3.3.1]nonane construction. Palladium-catalysed carbomethoxylation of the terminal alkene in 152 with Pd(dppf)Cl_2_, carbon monoxide, and methanol delivered the Michael acceptor precursor 153 in 85% yield.

The intramolecular Michael addition to forge the sterically congested bicyclo[3.3.1]nonane was further impeded by *syn*-pentane interactions with the axially disposed B-ring substituents. Nevertheless, treatment of 153 with KHMDS generated the reactive potassium enolate, which underwent the desired Michael addition to furnish the bridgehead-allylated analogue of 154 in 56% yield, with concomitant TMS deprotection. Removal of the allyl group was planned through alkene isomerization, oxidative cleavage, and decarboxylation. Palladium-catalysed alkene isomerization with PdCl_2_(MeCN)_2_ provided the internal alkene isomer iso-154 in 80% yield.^152^ Subjection of iso-154 to oxidative cleavage conditions developed by Borhan and co-workers resulted in smooth conversion,^153^ and after acidic aqueous workup with 3 M H_2_SO_4_, andirolide N (157) was obtained directly in 48% yield.

#### 4.4.2. Renata's total synthesis of mexicanolide (2025)

Mexicanolide (161) is the prototypical ring B,D-*seco* limonoid first isolated from the wood of *Cedrela odorata* in 1965.^154–156^ The most distinctive structural feature of mexicanolide is the simultaneous cleavage of the B and D rings, followed by rearrangement to a unique bicyclo[3.3.1]nonane framework bridged by a tetrahydrofuran ring. The ring B-*seco* rearrangement that defines the mexicanolide skeleton is believed to arise biosynthetically through oxidative fragmentation of the limonoid B-ring, followed by intramolecular 1,6-conjugate addition.^157,158^ This pathway was first investigated in biomimetic studies by Connolly and Ekong in the early 1970s,^148,149^ and was later exploited by Williams in the first total synthesis of mexicanolide in 2012.^159^

Building on the chemoenzymatic platform established in their 2022 total synthesis of gedunin,^61^ Renata and co-workers extended the strategy to mexicanolide (161) by exploiting the C7 hydroxyl group of a gedunin-type intermediate as a handle for skeletal rearrangement (Scheme 14).^60^ The synthesis commenced from deoxydeacetylgedunin (158), which was available in ten steps from sclareolide through the previously reported route (see Scheme 10 for details) or directly from commercial neem oil.^61^ Epoxidation of 158 with alkaline hydrogen peroxide proceeded in high yield with complete regio- and facial selectivity to give epoxyketone 159. The epoxide withstood the subsequent Suárez fragmentation, and Pinnick oxidation followed by methylation delivered the common intermediate 160 bearing both the B-ring-*seco* methyl ester and the A-ring epoxyketone.^160,161^

**Scheme 14:**
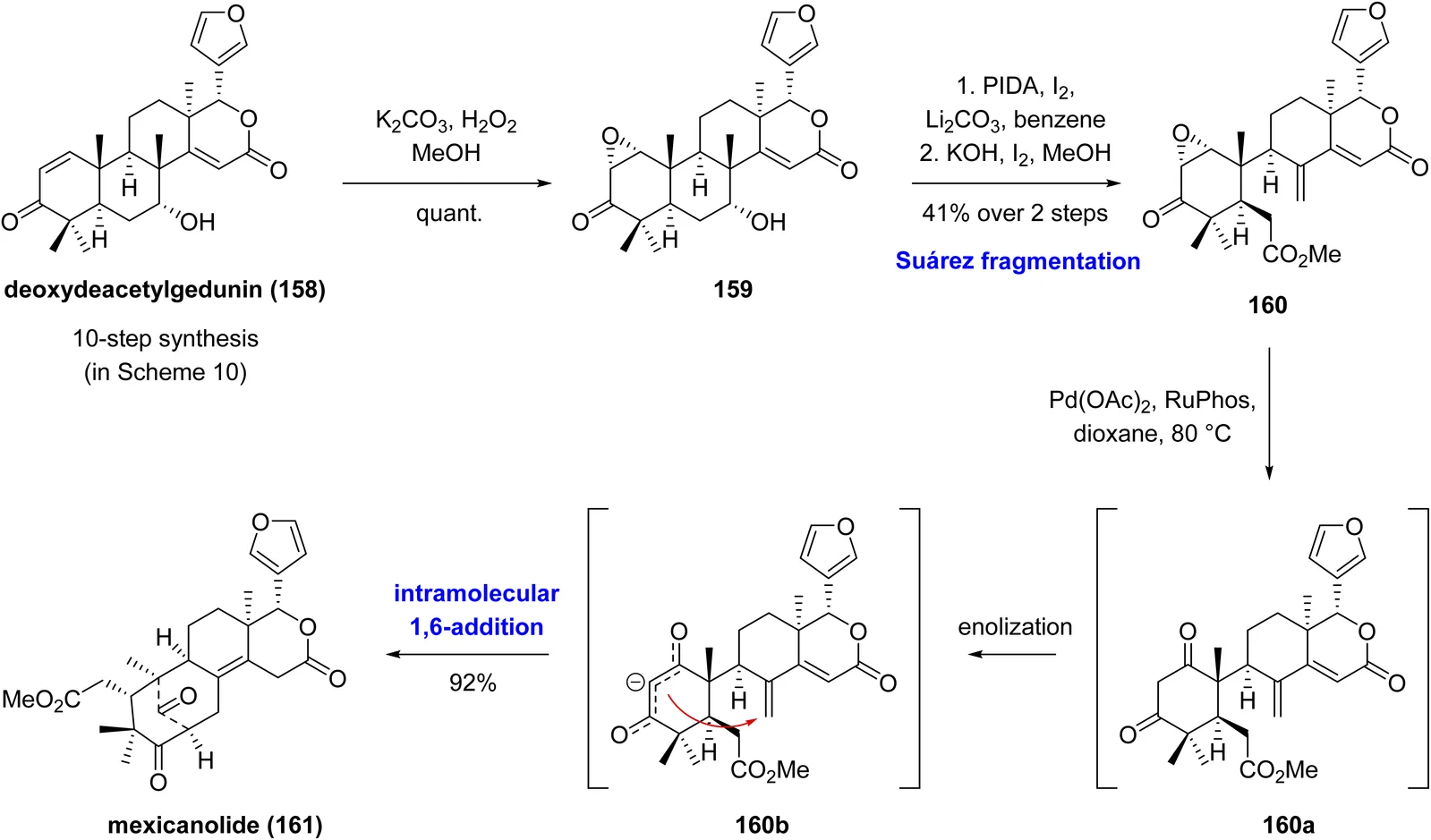
Renata's total synthesis of mexicanolide.^60^

With 160 in hand, a palladium-catalysed epoxyketone rearrangement generated a 1,6-dicarbonyl intermediate without forming the reactive C1 alcohol. Exposure of 160 to Pd(OAc)_2_ and RuPhos in dioxane at 80 °C promoted rearrangement to diketone 160a, which under the elevated temperature underwent enolization and intramolecular 1,6-conjugate addition (160b) to deliver mexicanolide (161) as the sole product in 92% yield.^162^ This concise conversion from deoxydeacetylgedunin (158) completed the total synthesis of mexicanolide (161) in 14 steps from sclareolide (5).

## 5. Total synthesis of rearranged limonoids

### 5.1 Yang and Chen's total synthesis of haperforin G (2020)

Haperforin G (181) is a trace limonoid first isolated from the roots of *Harrisonia perforata* by Khuong-Huu and co-workers,^163^ with subsequent isolation studies by Hao's group revealing its remarkable biological profile.^164^ As a potent inhibitor of human 11β-hydroxysteroid dehydrogenase type 1 (11β-HSD1) with an IC_50_ value of 0.58 µM, haperforin G has attracted considerable attention as a potential lead compound for the treatment of metabolic disorders including Alzheimer's disease, vascular inflammation, cardiovascular disease, and glaucoma.^165^ Structurally, haperforin G possesses a novel limonoid 6/5/6 tricyclic carbon skeleton bearing six stereogenic centers, including two all-carbon quaternary stereocenters at C10 and C13, two lactone moieties, and a 3-substituted furan ring. The most formidable synthetic challenges lie in the enantioselective construction of the two all-carbon quaternary carbons, the stereocontrolled installation of a tertiary chiral alcohol at C6, and the regioselective incorporation of both trisubstituted (C1–C2) and tetrasubstituted (C8–C14) double bonds.

In 2020, Yang and Chen's group reported the first total synthesis of haperforin G in 20 steps from commercially available starting materials (Scheme 15),^166,167^ leveraging a Co-catalysed intramolecular Pauson–Khand reaction and a visible-light photoredox-catalysed radical cross-coupling as the key strategic transformations. Retrosynthetically, the authors designed a convergent strategy wherein two advanced fragments, iodolactone 166 and enone 178, would be joined through the C11–C12 bond *via* a visible-light photoredox-catalysed alkyl radical coupling, followed by an intramolecular aldol reaction and dehydration to assemble the polycyclic core.

**Scheme 15:**
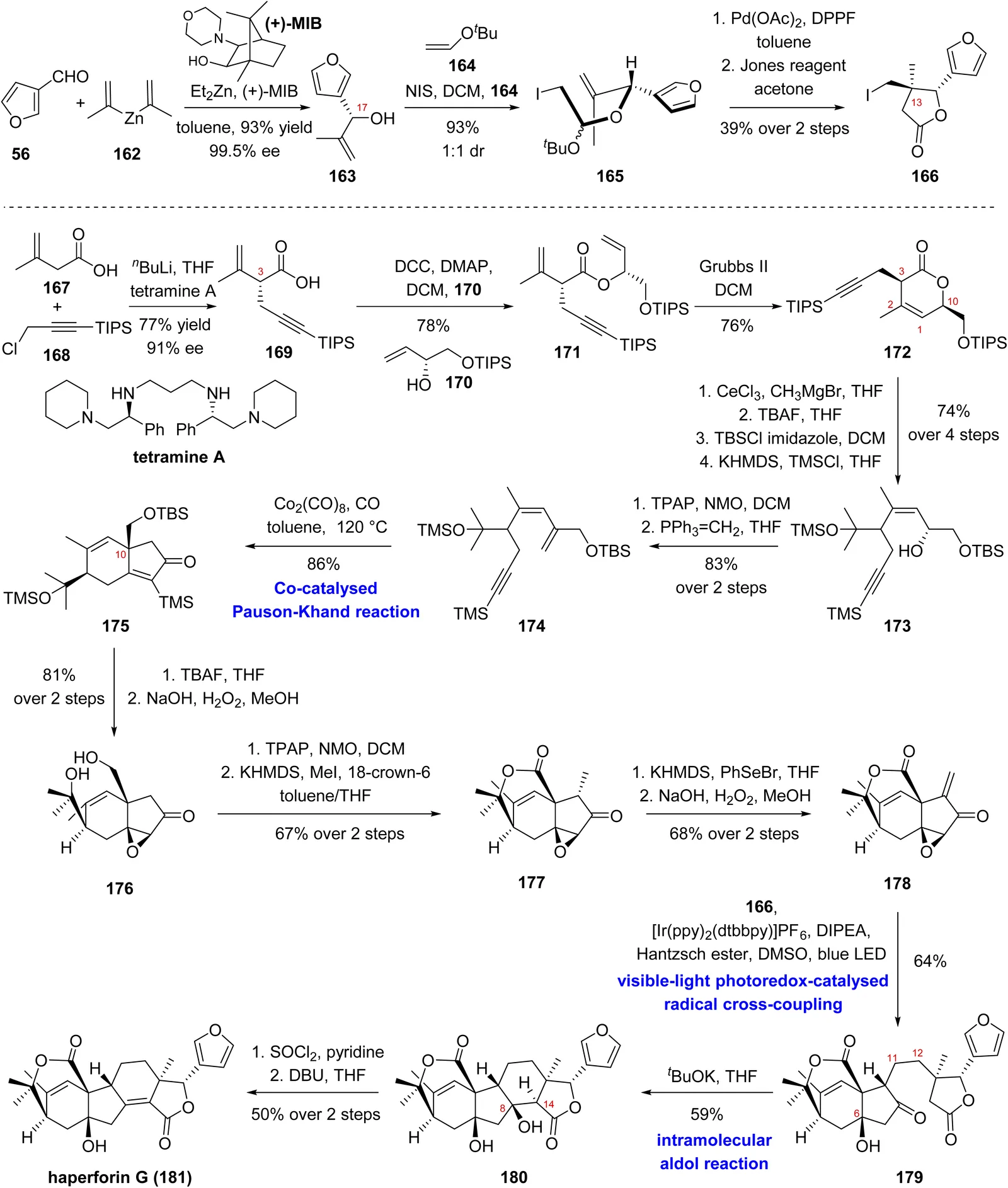
Yang and Chen's total synthesis of haperforin G.^166^

For the synthesis of iodolactone 166, aldehyde 56 was reacted with vinylzinc 162 under Nugent's (+)-MIB-mediated enantioselective vinylation conditions to produce allylic alcohol 163 bearing the desired C17 stereogenic center in 93% yield with excellent enantiomeric excess.^168^ NIS-mediated intermolecular ketalisation of 163 with *tert*-butyl vinyl ether provided iodide 165 in 93% yield.^169^ After optimisation, iodide 165 underwent a Pd-catalysed iodide atom transfer cyclisation, and subsequent Jones oxidation led to the formation of iodolactone 166 in 39% yield over two steps.^170^

With streamlined access to iodolactone 166 accomplished, attention turned to the synthesis of enone 178. Treatment of 3-methylbut-3-enoic acid (167) with ^*n*^BuLi in the presence of chiral tetramine A, followed by addition of chloride 168, furnished acid 169 bearing an (*R*)-configured C3 stereocenter in 77% yield and 91% ee.^171^ Esterification of 169 with alcohol 170 under DCC/DMAP conditions delivered diene 171 in 78% yield, which underwent a Grubbs II-catalysed diastereomer-discriminating ring-closing metathesis to produce lactone 172 as a single diastereoisomer in 76% yield.^172^ This concise three-step sequence established both the desired C3 and C10 stereogenic centers and the *cis*-trisubstituted olefin at C1–C2. Lactone 172 was then subjected to double addition of methylmagnesium bromide in the presence of CeCl_3_, followed by desilylation with TBAF, to afford triol. Sequential silylation and desilylation of triol provided 173 in 74% yield over four steps, and subsequent TPAP/NMO oxidation of the secondary alcohol followed by a Wittig reaction furnished enyne 174 in 83% yield over two steps, setting the stage for the pivotal Pauson–Khand cyclisation.

After extensive optimisation, exposure of enyne 174 to a catalytic amount of Co_2_(CO)_8_ (20 mol%) under a balloon pressure of CO at 120 °C for 36 h afforded the expected cyclopentenone 175 in 86% yield as a single diastereomer,^173^ thereby constructing the D-ring bearing the C10 all-carbon quaternary stereocenter. Treatment of 175 with TBAF followed by Weitz-Scheffer epoxidation with H_2_O_2_ in the presence of NaOH produced epoxide 176 in 81% overall yield in a regio- and diastereoselective manner. Gratifyingly, Ley oxidation of diol 176 directly converted it into the corresponding lactone, which was methylated with KHMDS and methyl iodide in the presence of 18-crown-6 to give 177 in 67% yield over two steps. Subsequent selenylation with KHMDS and PhSeBr, and oxidation with H_2_O_2_ in the presence of NaHCO_3_ delivered enone 178 in 68% yield over two steps.

With both key fragments in hand, the convergent coupling was investigated. Initial attempts using traditional AIBN/^*n*^Bu_3_SnH-mediated radical conditions resulted in low yields of the coupling product. Inspired by recent advances in photoredox catalysis,^174^ the authors found that treatment of enone 178 and iodide 166 with a catalytic amount of [Ir(ppy)_2_(dtbbpy)]PF_6_ under blue light irradiation, using a Hantzsch ester as the reducing agent and DIPEA as the base, converted the unactivated C(sp^3^)-based iodide 166 to its corresponding alkyl radical, which intermolecularly coupled with enone 178 to produce 179 as a single diastereoisomer in 64% yield. Treatment of 179 with ^*t*^BuOK effected an intramolecular aldol condensation to produce the polycyclic skeleton as a single stereoisomer in 59% yield (84% brsm). Finally, taking advantage of the hydroxyl group at C6, treatment of 180 with SOCl_2_ in pyridine generated an intermediate cyclic sulfate,^175^ which upon treatment with DBU afforded haperforin G (181) in 50% overall yield over two steps. The structure of the synthetic product was unequivocally confirmed by single-crystal X-ray analysis.^166,167^

### 5.2 Ding and Xuan's total synthesis of phragmalin and khayanolide-type limonoids (2024)

Moluccensins G (202) and H (203) are phragmalin-type limonoids isolated from the seeds of the Indian mangrove *Xylocarpus moluccensis*,^176,177^ characterised by a tricyclo[3.3.1. (ref. 2 and 10)1 (ref. 1 and 4)]decane carbon framework coupled with conjugated Δ^8,9^ and Δ^14,15^ double bonds. Two biogenetically relevant khayanolide-type limonoids featuring a tricyclo[4.2.1. (ref. 10 and 30)1 (ref. 1 and 4)]decane skeleton, namely krishnolide F (197) and khayseneganin F (199), were also identified from the same plant source and from *Khaya senegalensis*, respectively.^178,179^ Krishnolide F exhibits potent agonistic effects against the human pregnane X receptor (hPXR),^178^ while khayseneganin F stands out for its complex hexacyclic structure embedded with twelve consecutive stereogenic centers, including six quaternary centers, three of which are all-carbon and located at bridgehead positions. A putative biogenetic conversion from phragmalins to khayanolides was previously proposed by Nakatani,^180^ involving a strain-release driven C1(2 → 30) migration *via* either a radical pathway or a pinacol-type rearrangement, yet this hypothesis remained to be fully elucidated.

In 2024, Ding, Xuan, and co-workers reported the asymmetric and divergent total syntheses of all four natural products (Scheme 16),^181,182^ employing a torquoselective interrupted Nazarov cyclisation as the key step, together with a Liebeskind–Srogl coupling, a benzoin condensation, and bidirectional acyloin rearrangements. The synthesis commenced with the preparation of thioester 187 from commercially available (*S*)-(+)-10-methyl-1(9)-octal-2-one (182). Ketalisation of 182 in the presence of ethylene glycol and *p*-TsOH was accompanied by alkene migration to the β,γ-position. Upjohn dihydroxylation of the resulting olefin furnished diol 183 in 56% yield over two steps. Treatment of 183 with Ph_3_PCl_2_ induced a semipinacol rearrangement to afford *trans*-1-decalone in excellent yield. A one-pot enol triflation followed by CuI-catalysed cross coupling produced 184. Oxidative cleavage of the alkene 184 by ozonolysis generated an unstable keto-aldehyde, which was immediately treated with AcOH and piperidine for ring contraction to deliver 5/6 bicyclic aldehyde 185 with 12 : 1 dr at C5. NHC-catalysed oxidative thioesterification of 185 with triazolium salt as reported by Takemoto,^183^ afforded the corresponding thioester in 80% yield. After regioselective conversion to methyl enol ether 186 in 66% yield, sequential ozonolysis and acetalisation provided acetal thioester 187 in 58% yield.

**Scheme 16:**
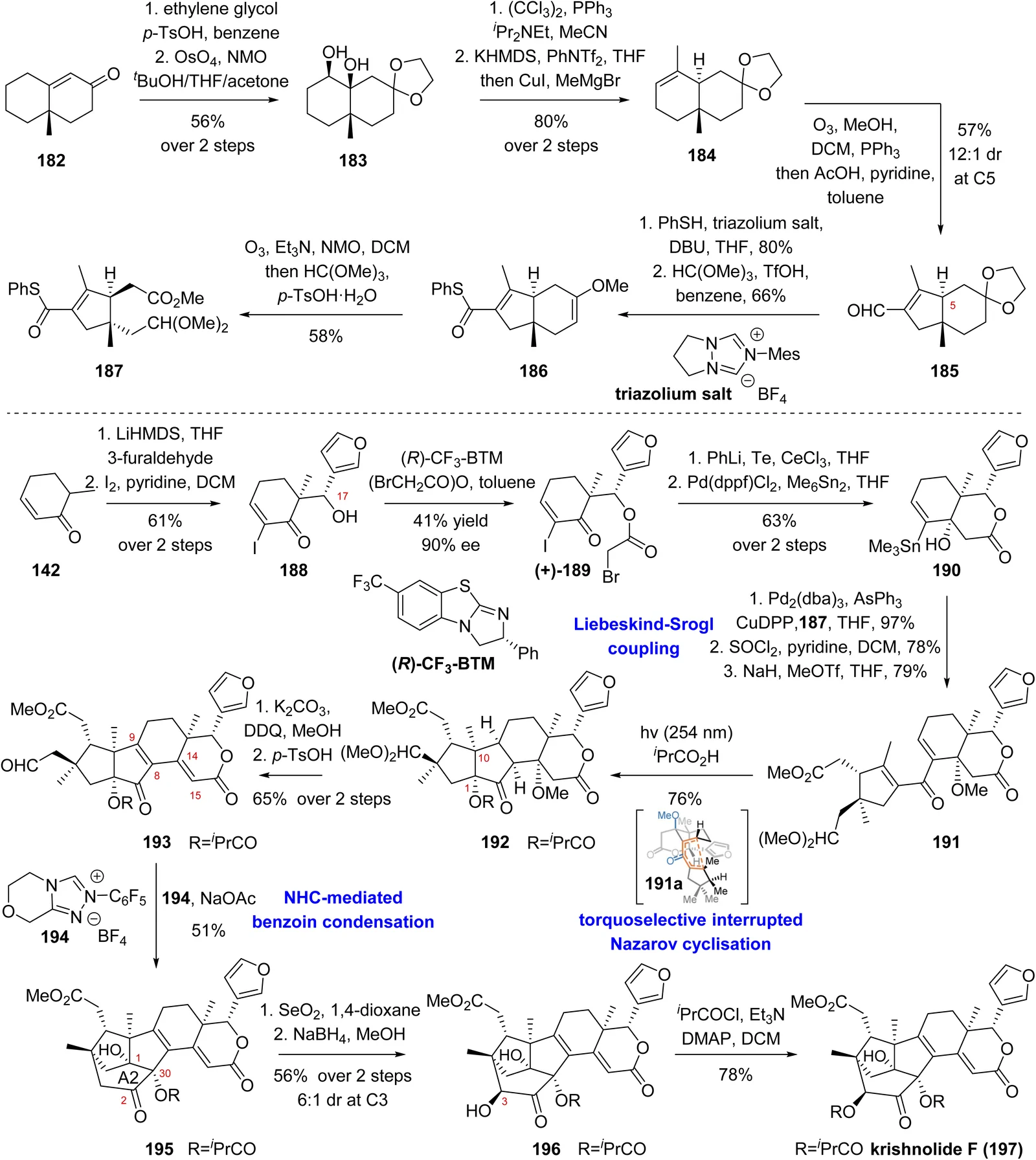
Ding and Xuan's total synthesis of krishnolide F.^181^ This figure has been adapted from ref. 181 with permission from the American Chemical Society, copyright 2025.

Meanwhile, vinyl stannane 190 was accessed from 6-methylcyclohex-2-en-1-one (142). A diastereoselective aldol reaction between 142 and 3-furaldehyde, followed by α-iodination, afforded α-iodoenone 188 in 61% yield over two steps. The acylative kinetic resolution of the benzylic alcohol at C17 was realised by the use of (*R*)-CF_3_-BTM and (BrCH_2_CO)_2_O, affording bromoacetate (+)-189 with excellent efficiency and enantioselectivity (41% yield, 90% ee). Subsequent lactone formation proved problematic under both Reformatsky-type and lithium–halogen exchange conditions, with low yields accompanied by substantial dehalogenation. Pleasingly, treatment with PhTeLi, prepared *in situ* from tellurium powder and phenyllithium, and CeCl_3_ effected the expected ring closure successfully.^184^ The resultant tertiary alcohol was then cross-coupled with Me_6_Sn_2_ to furnish vinyl stannane 190 in 63% yield over two steps.

With both fragments in hand, the Liebeskind–Srogl coupling in the presence of catalytic Pd_2_(dba)_3_/AsPh_3_ and a stoichiometric amount of copper(i) diphenylphosphinate was performed,^185^ smoothly providing desired product in 97% yield, which set the stage for the pivotal interrupted Nazarov cyclisation.^186^ Computational studies of the stereochemical preference revealed that an intramolecular hydrogen bond between the dienone carbonyl and the C14–OH stabilised the undesired cyclisation pathway, whereas masking the C14–OH as a methyl ether disrupted this interaction and reversed the torquoselectivity through strong *syn*-pentane dipole–dipole repulsion in the competing transition state.^181^ Accordingly, methyl ether 191, prepared from the coupling product *via* methylation in 79% yield, was identified as the optimal substrate for the photo-induced interrupted Nazarov cyclisation.^187^ Upon UV irradiation in the presence of ^*i*^PrCO_2_H, 191 delivered the anticipated product 192 in 76% yield as a single diastereomer, with interception of the oxyallyl cation occurring at the less encumbered position to furnish the C1β-tertiary alcohol.

Encouraged by these results, the authors turned to acquiring common intermediate 196. Base-promoted elimination followed by oxidative dehydrogenation of 192 with K_2_CO_3_ and DDQ was executed in one pot, forging the conjugated Δ,^8,9^Δ^14,15^-diene. Treatment with *p*-TsOH to release the aldehyde moiety afforded keto-aldehyde 193 in 65% yield over two steps. The remaining challenge in construction of the tricyclo[4.2.1. (ref. 10 and 30)1 (ref. 1 and 4)]decane core required creation of the C2–C30 bond to form the A2 ring. After extensive screening, the NHC-mediated benzoin condensation using triazolium salt 194 and NaOAc proved most effective, and rigorous degassing of the reaction mixture to avoid superfluous oxidation of the air-sensitive Breslow intermediate provided pentacycle 195 in 51% yield. The spontaneous isobutyryl transfer from C1 to the C30-oxygen atom also played a critical role in driving the conversion of the Breslow intermediate to 195.

Having secured sufficient quantities of 195, the authors focused on the late-stage syntheses of the four target natural products. For the khayanolide series, the next task was to install an α-hydroxyl group at the C3 position of 196. Exhaustive attempts at direct α-hydroxylation were unsuccessful, providing exclusive β-oriented stereoselectivity. As an alternative solution, Riley oxidation of 195 with SeO_2_ afforded the α-diketone, which then underwent a regio- and diastereoselective reduction in the presence of NaBH_4_ to provide 196 in 56% yield (6 : 1 dr at C3) over two steps. Ensuing acylation of the newly formed C3–OH proceeded efficiently and delivered krishnolide F (197) in 78% yield.

Further elaboration to khayseneganin F (199) necessitated site-selective deoxygenation at C30 and 1,6-reduction of the dienoate moiety. Inspired by López's work,^188^ a one-pot sulfa-conjugate addition/desulfurisation cascade process employing PhSH, AIBN, and ^*n*^Bu_3_SnH was applied on the TMS ether of 196, successfully achieving the 1,4-reduction product 198 with concurrent removal of the isobutyrate in 52% yield over two steps with a 2.3 : 1 mixture of Δ^8,14^ and Δ^8,9^-monoalkenes. After stereoselective installation of the C8β-OH, a NaOMe-promoted C2-ketalisation/oxa-Michael sequence proceeded uneventfully to furnish khayseneganin F (199) in 58% yield over two steps (Scheme 17).

**Scheme 17:**
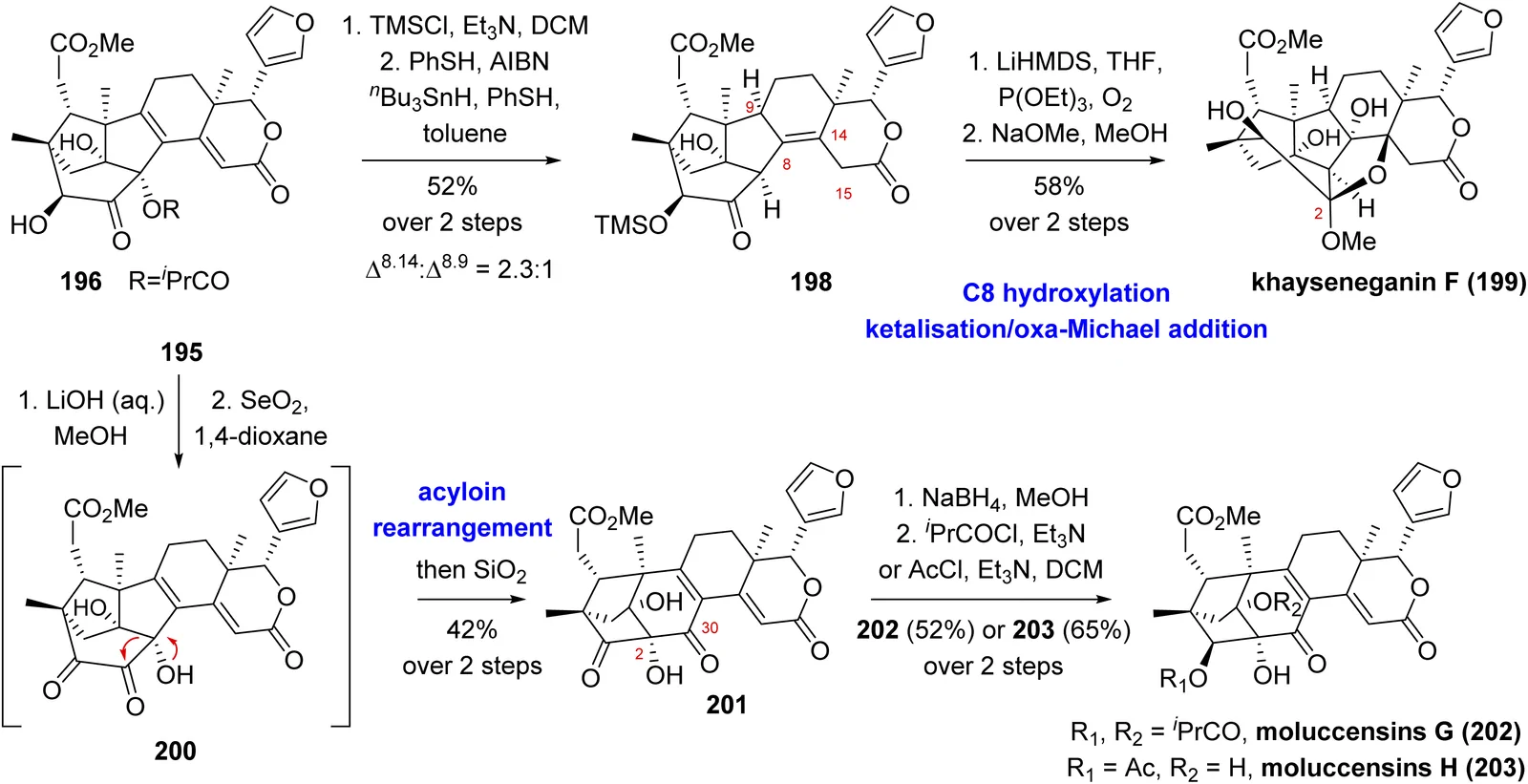
Ding and Xuan's total synthesis of khayseneganin F, moluccensins G, moluccensins H.^181^

On the other hand, the phragmalin series was accessed through a distinct pathway. Sequential saponification and Riley oxidation of 195 gave rise to an unstable α-ketol 200. During silica gel column chromatography, the expected C1(30 → 2) acyloin rearrangement occurred spontaneously, affording 201 in 42% yield over two steps. Eventually, by applying a two-step protocol involving stereoselective reduction and acylation, moluccensins G (202) and H (203) were obtained in 52% and 65% yield, respectively (Scheme 17). Furthermore, the interconversion between moluccensins G (202) and krishnolide F (197) was explored. Exposing 202 to 4-DMAP at room temperature resulted in a smooth C1(2 → 30) acyloin rearrangement to provide a 1 : 4 mixture of 202 and 197 in 94% combined yield, whereas the similar phenomenon was not observed for the same treatment of 197.

### 5.3 Newhouse's total synthesis of phragmalin-type limonoids (2025)

Thaigranatin P (221) is a phragmalin-type limonoid isolated from the Thai mangrove plant (*Xylocarpus granatum*), first reported by Shen's group in 2021.^189^ It features the signature cage-like tricyclo[3.3.1. (ref. 2 and 10)1 (ref. 1 and 4)]decane core characteristic of phragmalin natural products but notably lacks the typical C2 oxidation state present in most congeners. Biologically, thaigranatin P showed an agonistic effect on the human pregnane X receptor (hPXR) at a concentration of 10 nM,^189^ highlighting its potential as a lead compound for modulating xenobiotic metabolism.

In 2025, Newhouse's group reported a convergent total synthesis of thaigranatin P (Scheme 18),^190^ featuring a SmI_2_-mediated reductive coupling of an aldehyde and an alkene as the key transformation for constructing the signature tricyclo[3.3.1. (ref. 2 and 10)1 (ref. 1 and 4)]decane framework, together with a strategically convergent assembly of the phragmalin core *via* a cuprate addition, triflation, and intramolecular Heck reaction sequence. In addition to thaigranatin P, they also accomplished the total syntheses of erythrocarpines M and L and the putative natural product thaigranatin V,^190^ all of which are architecturally complex phragmalin-type limonoids.

**Scheme 18:**
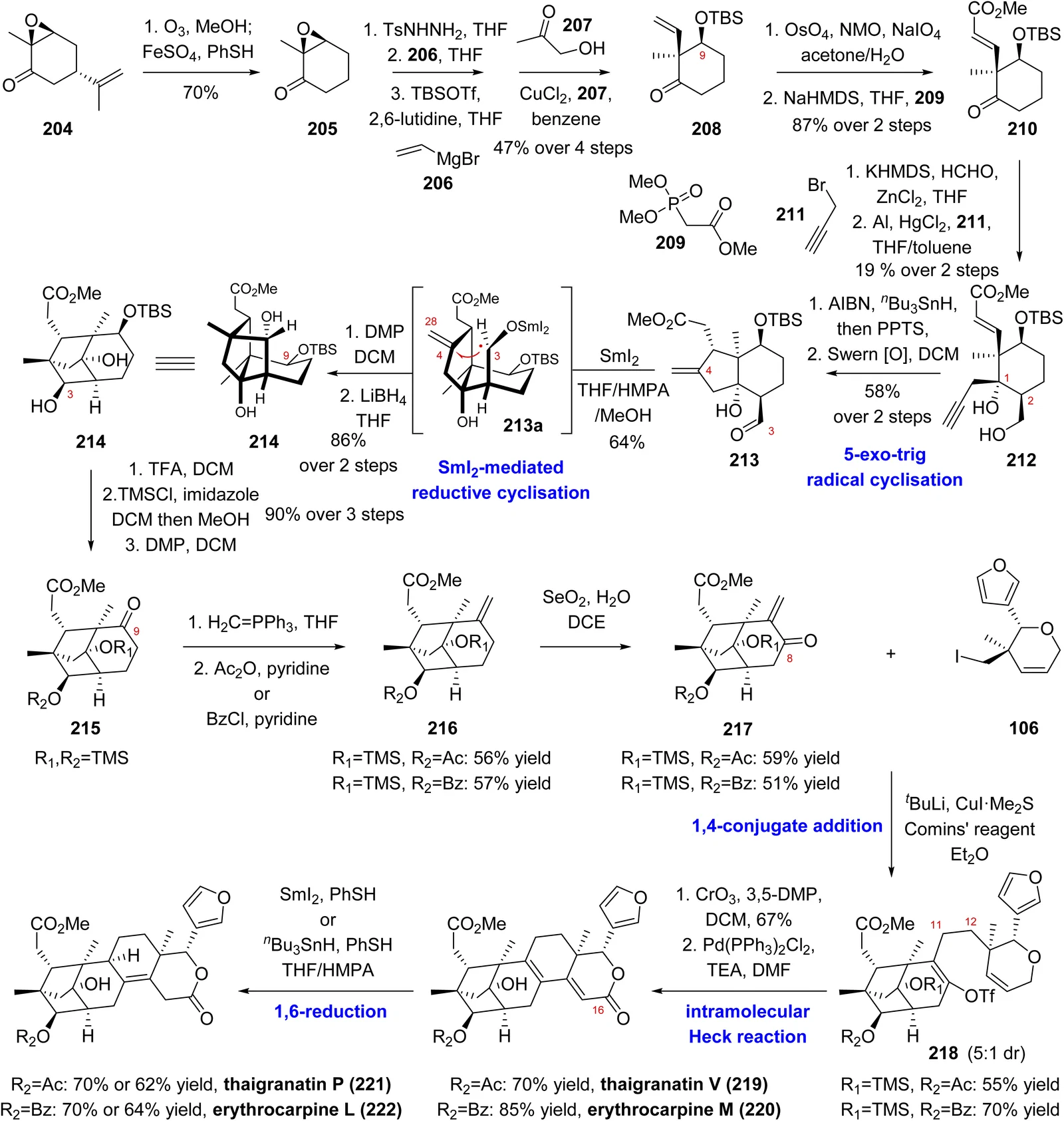
Newhouse's total synthesis of phragmalin-type limonoids.^190^

The asymmetric synthesis of the west fragment 217 commenced with the known (*S*)-carvone monoxide 204. The isopropenyl side chain was selectively cleaved using Kwon's hydrodealkenylative fragmentation protocol to give enantioenriched epoxide 205 in 70% yield.^191^ Epoxide 205 was then subjected to syn opening with vinylmagnesium bromide, in which the tosylhydrazone auxiliary governed regioselectivity and the liberated alkoxide directed the stereochemistry.^192^ Subsequent TBS protection of the secondary alcohol and removal of the hydrazone group afforded a terminal vinyl intermediate 208 in 47% yield over four steps. This olefin underwent oxidative cleavage to give aldehyde and was converted to the desired methyl ester (+)-210 in 93% yield *via* a Horner–Wadsworth–Emmons olefination sequence.

With enantioenriched 210 in hand, the aldol reaction to freshly depolymerized formaldehyde furnished the desired product (36%) with its C2-epimer as the major product (54%), reflecting a slight preference for equatorial attack at C2. Fortunately, the undesired C2-epimer could be efficiently recycled to the desired product through enolization with imidazole in 37% yield (99% brsm).^193^ Subsequent addition of a propargyl aluminium reagent to the ketone provided 212 in 19% yield over two steps along with the undesired diastereomer at C1 in 26% yield.^194^ Compound 212 underwent a radical cyclisation to forge the *cis*-fused 5,6-bicyclic system. The C3 primary alcohol was oxidized to the corresponding aldehyde 213 in 58% yield over two steps, which was subjected to SmI_2_-mediated reductive cyclisation to establish the core tricyclic framework in 64% yield.^195^ Reaction temperature control was essential to suppress competing reduction and undesired cyclisation involving the alkyl samarium species and the methyl ester. The stereochemistry at C3 was proposed to arise from an intermediate in which the sterically bulky samarium alkoxide is oriented away from the cage structure (213a). The desired stereochemistry at C3 was secured *via* an oxidation–reduction sequence to afford intermediate 214 in 86% yield.

A one-pot TBS deprotection, global TMS protection, and selective C9 deprotection sequence was performed, and the resulting C9 alcohol was oxidized to ketone 215 in 90% yield. A salt-free Wittig reaction accompanied by concurrent TMS deprotection at the C3 alcohol and esterification of the liberated alcohol provided 216-Ac and 216-Bz in 56% and 57% yields, respectively. Allylic oxidation at C8 provided the exocyclic enones 217-Ac and 217-Bz in 59% and 51% yields, respectively. Notably, the addition of a superstoichiometric amount of water was crucial to ensure reproducibility and high yield in this transformation.^196^ Meanwhile, the preparation of enantiopure east fragment alkyl iodide 106 could be achieved by using the Renata group's method in the total synthesis of gedunin (see Scheme 10 for details).^60,61^

With both fragments in hand, the C11–C12 bond was formed through a 1,4-conjugate addition of a Gilman reagent, prepared *in situ* from alkyl iodide 106, to enantioenriched 217-Ac and 217-Bz. The corresponding enolate was trapped with Comins' reagent to afford the vinyl triflates 218-Ac and 218-Bz in 55% and 70% yields, respectively.^197^ Oxidation at C16 was achieved with CrO_3_ and 3,5-dimethylpyrazole in 67% yield.^198^ The critical intramolecular Heck reaction proceeded smoothly using Pd(PPh_3_)_2_Cl_2_ to yield the putative natural product thaigranatin V (219) in 70% yield and the natural product erythrocarpine M (220) in 85% yield.^199^ Other ligand systems displayed either poor conversion or significant protodehalogenation. The reported 1,6-reduction with PhSH, ^*n*^Bu_3_SnH, and AIBN proceeded smoothly to afford thaigranatin P (221) and erythrocarpine L (222). The authors also found that SmI_2_ and PhSH could enable a selective 1,6-reduction in good yield,^181^ while the use of a proton source such as MeOH led to exclusive formation of an undesired 1,4-reduction product.

## 6. Total synthesis of limonoid alkaloids

### 6.1 Newhouse's total synthesis of xylogranatopyridine B (2018)

Xylogranatopyridine B (237) is a trace isolate from the leaves of the Chinese mangrove (*Xylocarpus granatum*) and belongs to a recently discovered class of limonoid alkaloids.^200^ The defining structural feature of 237 is a tetrasubstituted pyridine ring embedded at the core of a tetracyclic limonoid framework bearing a furan-fused γ-lactone D-ring.

In 2018, Newhouse and co-workers reported the first asymmetric total synthesis of 237 in 11 steps from commercially available dihydrocarvone (Scheme 19).^201,202^ The synthetic strategy employed a late-stage convergent fragment coupling through a modified Liebeskind pyridine synthesis.^203^ The achiral oxime benzoate fragment 226 was prepared from 3-methyl-2-cyclohexenone 223*via* methylcuprate conjugate addition and TMSCl trapping to furnish enoxysilane 224. Oxidation of 224 under Yamamoto conditions with *in situ* generated TIPS-NO_2_ afforded α-keto oxime 225 in 63% yield over two steps.^204^ Wittig methylenation followed by benzoylation with benzoic anhydride completed fragment 226 in 62% yield over two steps.

**Scheme 19:**
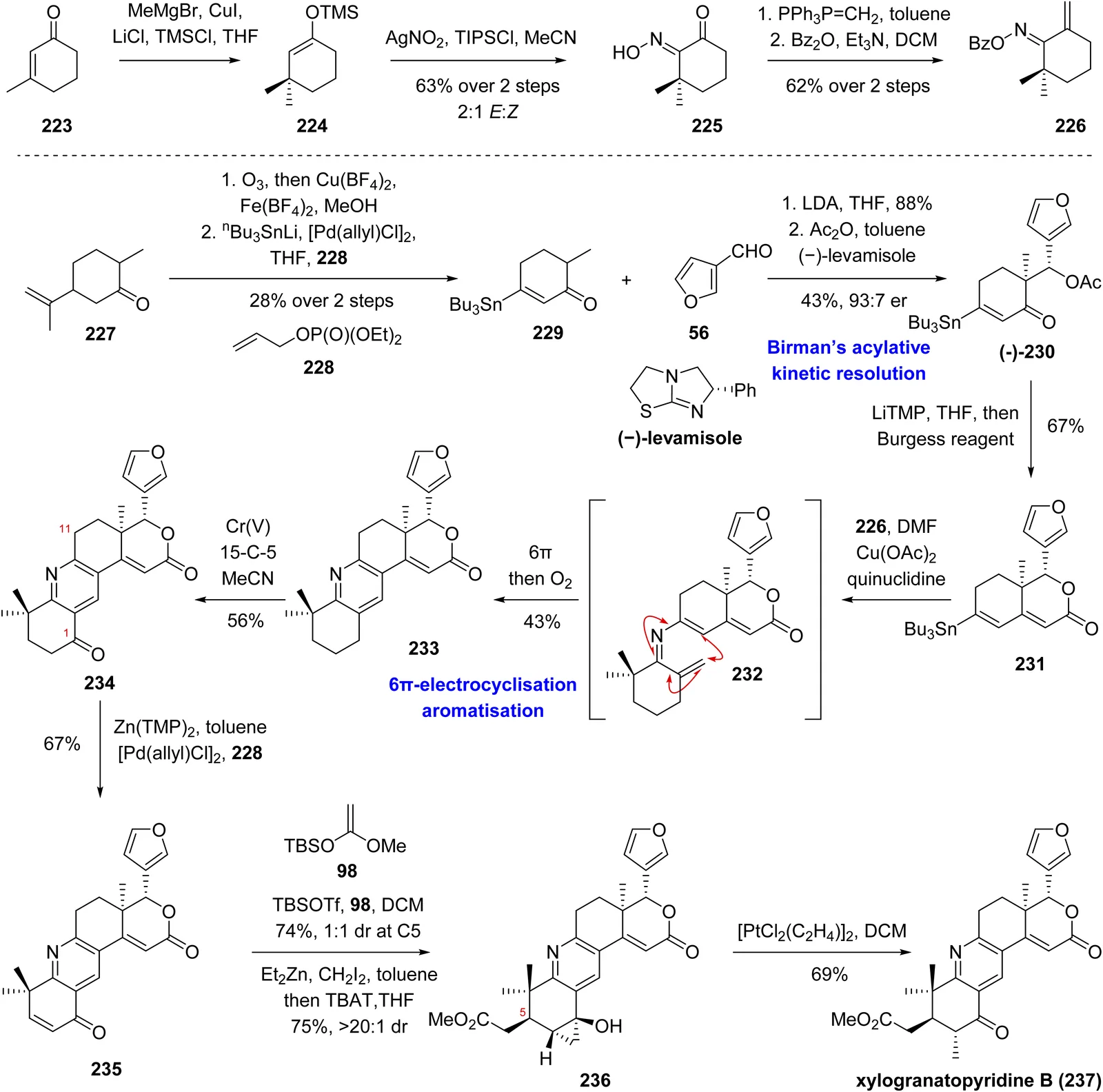
Newhouse's total synthesis of xylogranatopyridine B.^201^

The stannane fragment 231 was derived from dihydrocarvone 227. Ozonolytic fragmentation of 227 provided 6-methylcyclohexenone,^147^ and subsequent palladium-catalysed allylic oxidative β-stannylation using tributylstannyllithium and diethyl allyl phosphate furnished β-stannyl enone 229 in 28% yield over two steps on a multigram scale. Diastereoselective aldol addition of the lithium enolate of 229 to 3-furaldehyde 56 gave the racemic alcohol. The racemic synthesis was rendered asymmetric through Birman's acylative kinetic resolution by using (−)-levamisole, which furnished acetate (−)-230 in 93 : 7 er and 43% yield on a decagram scale. Treatment of (−)-230 with LiTMP initiated an intramolecular acetate aldol reaction, and subsequent dehydration with Burgess reagent provided stannyl dienoate 231 in 67% yield.

The key fragment coupling between oxime 226 and stannane 231 was promoted by stoichiometric Cu(OAc)_2_ with quinuclidine as an additive in DMF at 60 °C. This Chan-Lam-type C–N bond formation was followed by a thermal 6π-electrocyclisation and aromatisation cascade that assembled the tetracyclic pyridine core 233 in 43% yield.^205,206^ Site-selective benzylic oxidation at the more electron-rich C1 position was accomplished with a Cr(v) complex to give ketone 234 in 56% yield with 27% recovered 233.^207^ Palladium-catalysed α,β-dehydrogenation of 234 provided enone 235 in 67% yield.^208^ A Mukaiyama–Michael reaction of 235 with the TBS silyl ketene acetal 98 derived from methyl acetate in the presence of catalytic TBSOTf installed the ester side chain as a 1 : 1 mixture of C5 diastereomers. Simmons-Smith cyclopropanation with Et_2_Zn and CH_2_I_2_ followed by TBAT-mediated deprotection of the siloxycyclopropane proceeded with high diastereoselectivity (>20 : 1 dr) to afford cyclopropanol 236 in 75% combined yield for the C5 epimers. Finally, platinum-catalysed ring opening of 236 with Zeise's dimer furnished xylogranatopyridine B (237) in 69% yield.^209^

### 6.2 Newhouse's total synthesis of granatumine A (2019)

Like xylogranatopyridine B, granatumine A (256) is a bislactone limonoid alkaloid isolated from the Chinese mangrove (*Xylocarpus granatum*).^200^ It exhibits moderate inhibitory activity against protein tyrosine phosphatase 1B, whereas the structurally related xylogranatopyridine B was found to be inactive.^200^ This enhanced potency has been attributed to the acid-labile C3 benzylic ether substituent and the reorganised A-ring bearing a fused lactone.

In 2019, Newhouse and co-workers achieved the first asymmetric total synthesis of granatumine A in 10 steps (Scheme 20).^202,210^ The synthesis employed a convergent fragment coupling approach in which a Knoevenagel condensation followed by carbonyl-selective oxa-6π-electrocyclisation forged a 2*H*-pyran precursor to the central pyridine.^211^

**Scheme 20:**
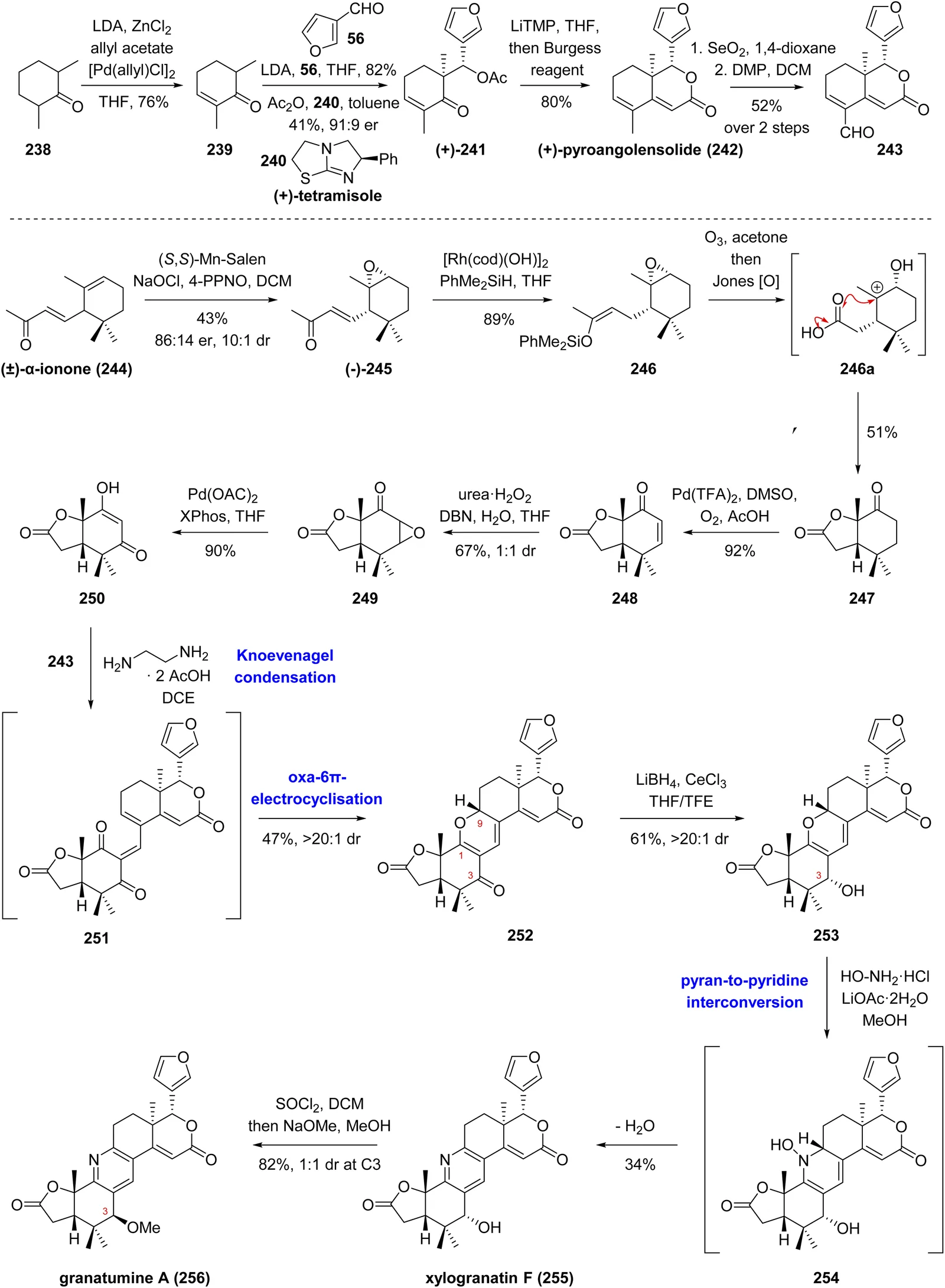
Newhouse's total synthesis of granatumine A.^210^

The aldehyde fragment 243 was synthesized from 2,6-dimethylcyclohexanone (238). Palladium-catalysed ketone dehydrogenation using inexpensive allyl acetate as the oxidant and LDA as the base converted 238 to enone 239 in 76% yield.^208^ A diastereoselective aldol reaction of 239 with 3-furaldehyde (56) afforded the corresponding alcohol in 82% yield.^212^ Acylative kinetic resolution with (+)-tetramisole (240) provided acetate (+)-241 in 41% yield and 91 : 9 er with 46% recovered starting material.^213^ The unreacted enantiomer could be recycled to 239 by retro-aldol in 85% yield, affording a 57% overall yield of (+)-241 after one recovery cycle. Treatment of (+)-241 with LiHMDS triggered an intramolecular aldol reaction, and dehydration with Burgess reagent delivered (+)-pyroangolensolide (242) in 80% yield, marking its first enantioselective synthesis.^214^ Site-selective allylic oxidation of 242 with SeO_2_ and Na_2_HPO_4_ followed by Dess–Martin oxidation furnished aldehyde 243 in 52% yield over two steps with 27% recovered 242.

The 1,3-diketone fragment 250 was prepared from (±)-α-ionone (244). Direct kinetic resolution *via* Jacobsen (*S*,*S*)–Mn-Salen catalysed epoxidation using buffered bleach as the terminal oxidant provided (−)-epoxy-α-ionone (245) in 43% yield, 86 : 14 er, and 10 : 1 dr.^215^ Rhodium-catalysed hydrosilylation of the enone moiety with [Rh(cod)(OH)]_2_ and phenyldimethylsilane gave enoxysilane 246 in 89% yield on a decagram scale.^216^ Ozonolytic cleavage of 246 followed by Jones oxidation afforded ketone 247 in 51% yield, presumably through carboxylic acid 246a which cyclised onto the tertiary carbocation generated by acid-mediated epoxide opening. Stahl's palladium-catalysed α,β-dehydrogenation converted 247 to enone 248.^217^ Nucleophilic epoxidation of 248 with urea hydrogen peroxide and DBN produced epoxide 249 in 67% yield as a 1 : 1 mixture of diastereomers. Palladium-catalysed rearrangement of 249 using Pd(OAc)_2_ and XPhos delivered 1,3-diketone 250 in 90% yield.^218^

The convergent assembly commenced with a Knoevenagel condensation of 250 and aldehyde 243 mediated by ethylenediammonium diacetate. The resulting enedione intermediate 251 underwent a spontaneous thermal oxa-6π-electrocyclisation to furnish 2*H*-pyran 252 as a single regioisomer in 47% yield on a gram scale.^219,220^ Luche reduction of the C3 ketone with LiBH_4_ and CeCl_3_ in trifluoroethanol provided allylic alcohol 253 in 61% yield as a single diastereomer. Treatment of 253 with hydroxylamine hydrochloride in refluxing methanol effected a pyran-to-pyridine interconversion, yielding the originally proposed structure of xylogranatin F (255) in 34% yield. The mismatch between the spectroscopic data of synthetic 255 and those reported for the natural product confirmed the misassignment of xylogranatin F.^221,222^ To complete the synthesis of granatumine A (256), the benzylic alcohol in 255 was chlorinated with thionyl chloride and subjected to *in situ* methanolysis, affording 256 as a separable 1 : 1 mixture of diastereomers in 82% yield.

## 7. Conclusion and outlook

The past decade has witnessed a paradigm shift in limonoid total synthesis, driven by the convergence of classical synthetic ingenuity with modern catalytic and biocatalytic tools. Linear, step-intensive routes have been largely supplanted by convergent fragment-coupling strategies, with biocatalytic C–H oxidation and radical-based bond construction emerging as the most impactful enabling technologies. These advances have substantially shortened synthetic routes and enabled access to increasingly complex limonoid scaffolds (Table 1). Despite these advances, significant challenges remain. The majority of naturally occurring limonoids, particularly those with highly rearranged carbon frameworks and complex oxygenation patterns, have yet to be achieved by total synthesis. Scalability continues to be a persistent concern, and the biological evaluation of synthetic limonoids and their analogues lags behind synthetic progress. Looking forward, the continued expansion of chemoenzymatic platforms and the development of divergent synthesis strategies capable of delivering multiple limonoid skeletons from common intermediates are expected to further broaden the scope and efficiency of the field. Ultimately, the convergence of synthetic chemistry and biocatalysis positions limonoid synthesis for continued breakthroughs in the decade ahead, facilitating deeper exploration of their biological functions and therapeutic potential.

**Table 1:** Summary of total synthesis of limonoids reported from 2017 to 2026

Classes	Representative natural products	Key strategies	Group (Year)	Ref.
Ring-intact limonoids	Azadiradione	Mukaiyama-aldol coupling; Pd-catalysed intramolecular Heck cyclisation	Ma (2025)	53
	Azadiradione	Chemoenzymatic hydroxylation; Luche reductive coupling; HAT-based Giese cyclisation	Renata (2025)	60
	Azadiradione	Iterative Robinson annulation; sterically assisted hydroboration/boron chain-walking/oxidation	Dethe (2026)	75
Ring C-*seco* limonoids	Nimbolide	Gold-catalysed Rautenstrauch rearrangement; etherification/5-*exo*-trig radical cyclisation	Qin & Yu (2023)	84
	Nimbolide	Chemoenzymatic hydroxylation; Suárez fragmentation; etherification/5-*exo*-trig radical cyclisation	Li (2024)	94
	Nimbolide	Asymmetric intermolecular Diels–Alder reaction; Pd-catalysed reductive Heck reaction; etherification/5-*exo*-trig radical cyclisation	Ma (2024)	102
	Nimbolide	Pd-catalysed borylative Heck cyclisation; etherification/5-*exo*-trig radical cyclisation	Sarlah (2025)	106
Ring D-*seco* limonoids	Gedunin	Chemoenzymatic hydroxylation; Luche reductive coupling; HAT-based Giese cyclisation	Renata (2022)	61
Ring A,D-*seco* limonoids	Deoxylimonin	Radical polyene cyclisation; Baldwin–Sanford oxidation; TOMA cascade reaction	Gui (2026)	125
	Deoxylimonin	Local computational models; intramolecular Diels–Alder reaction; oxa-Michael cyclisation	Newhouse (2026)	136
Ring B,D-*seco* limonoids	Andirolide N	Intermolecular Diels–Alder reaction; intramolecular Michael addition	Newhouse (2017)	146
	Mexicanolide	Chemoenzymatic hydroxylation; Suárez fragmentation; Pd-catalysed epoxyketone rearrangement; intramolecular 1,6-conjugate addition	Renata (2025)	60
Rearranged limonoids	Haperforin G	Co-catalysed intramolecular Pauson–Khand; visible-light photoredox radical cross-coupling; intramolecular aldol reaction	Yang & Chen (2020)	166
	Krishnolide F	Torquoselective interrupted Nazarov cyclisation; Liebeskind–Srogl coupling; NHC-catalysed benzoin condensation; bidirectional acyloin rearrangement	Ding & Xuan (2024)	181
	Khayseneganin F			
	Moluccensin G			
	Moluccensin H			
	Thaigranatin P	SmI_2_-mediated reductive cyclisation; intramolecular Heck; 1,4-conjugate addition; Kwon hydrodealkenylative fragmentation	Newhouse (2025)	190
	Erythrocarpine M			
	Erythrocarpine L			
	Thaigranatin V			
Limonoid alkaloids	Xylogranatopyridine B	Modified Liebeskind pyridine synthesis; 6π-electrocyclisation/aromatisation cascade; Birman's acylative kinetic resolution	Newhouse (2018)	201
	Granatumine A	Knoevenagel/oxa-6π-electrocyclisation cascade; pyran-to-pyridine interconversion; Birman's acylative kinetic resolution	Newhouse (2019)	210

## Author contributions

Wei Chen: writing – original draft, writing – review & editing. Xiaobo Zhou: investigation, writing – original draft. Dongsheng Yang: supervision, funding acquisition. All authors have read and agreed to the published version of the manuscript.

## Conflicts of interest

There are no conflicts to declare.

## Data Availability

No primary research results, software or code have been included and no new data were generated or analysed as part of this review.
